# RNA localization during early development of the axolotl

**DOI:** 10.3389/fcell.2023.1260795

**Published:** 2023-10-19

**Authors:** Kateřina Šimková, Ravindra Naraine, Jan Vintr, Vladimír Soukup, Radek Šindelka

**Affiliations:** ^1^ Laboratory of Gene Expression, Institute of Biotechnology of the Czech Academy of Sciences, Vestec, Czechia; ^2^ Department of Zoology, Faculty of Science, Charles University, Prague, Czechia

**Keywords:** RNA localization, early development, *Ambystoma mexicanum*, animal-vegetal axis, TOMO-seq

## Abstract

The asymmetric localization of biomolecules is critical for body plan development. One of the most popular model organisms for early embryogenesis studies is *Xenopus laevis* but there is a lack of information in other animal species. Here, we compared the early development of two amphibian species—the frog *X. laevis* and the axolotl *Ambystoma mexicanum*. This study aimed to identify asymmetrically localized RNAs along the animal-vegetal axis during the early development of *A. mexicanum*. For that purpose, we performed spatial transcriptome-wide analysis at low resolution, which revealed dynamic changes along the animal-vegetal axis classified into the following categories: profile alteration, *de novo* synthesis and degradation. Surprisingly, our results showed that many of the vegetally localized genes, which are important for germ cell development, are degraded during early development. Furthermore, we assessed the motif presence in UTRs of degraded mRNAs and revealed the enrichment of several motifs in RNAs of germ cell markers. Our results suggest novel reorganization of the transcriptome during embryogenesis of *A. mexicanum* to converge to the similar developmental pattern as the *X. laevis*.

## Introduction

Asymmetric distribution of biomolecules and asymmetric cell division is a crucial mechanism during stem cell division and the development of body tissues and internal organs. Moreover, it plays a critical role in the early development of many animal species. Maternal determinants such as proteins and RNAs (coding and non-coding), are asymmetrically distributed within the oocyte and early embryos. Thus, cell division often results in two unequal daughter cells with distinct fates. This phenomenon has been observed mainly in fish—*Danio rerio* (Howley and Ho, 2000)—and anuran amphibians—*Xenopus laevis* (Forristall et al., 1995; Kloc and Etkin, 1995; Sindelka et al., 2018; 2010) or *Rana pipiens* (Nath et al., 2005)—but interestingly there is no evidence of determinants localization in early mammalian embryos (Vinot et al., 2005). Likewise, our knowledge on the early development of other non-mammalian vertebrates, such as urodeles amphibians is still limited. In one of these urodeles, the Mexican axolotl (*Ambystoma mexicanum*), the maternal asymmetrical localization of several genes during the early development has been described (Vaur et al., 2003; Bachvarova et al., 2004). However, the whole transcriptome analysis, which could have the potential to reveal the similarities and differences between embryos of two close amphibian orders, is still missing. Therefore, we performed the comparison of RNA localization during the early development of the frog *X. laevis* (order *Anura*) and the axolotl *A. mexicanum* (order *Urodela*).

The anuran amphibian, like *X. laevis*, lays a copious number of large sized eggs (∼1.3 mm) which show a clear delineation of two hemispheres. The animal hemisphere is typically dark due to pigment granules and contains the germinal vesicle, while the vegetal hemisphere is light and full of yolk proteins and contains important organelles such as the endoplasmic reticulum, Golgi apparatus and mitochondria (Dumont, 1972). Eggs of urodele amphibians, like *A. mexicanum,* are typically larger (∼2 mm) and also show a clear animal and vegetal hemisphere (Schreckenberg and Jacobson, 1975; Bordzilovskaya and Dettlaff, 1979). In amphibians, the hemispheric distinction occurs during oogenesis, and simultaneously asymmetric distribution of maternal RNAs and proteins is established. The gradient formation of maternal molecules is the first step in the establishment of the animal-vegetal (A-V) axis, which is important for the development of the germ layers. In *X. laevis,* the blastomere fate mapping shows that the animal part of the embryo contributes to the ectoderm formation, the vegetal part into endoderm structures and the equatorial segment into mesodermal structures (Moody, 1987a; Moody, 1987b). To study Urodeles gastrulation, cell lineage tracing was performed in *A. mexicanum* (Lundmark, 1986), but comparatively thorough blastomere fate mapping of early embryos has never been done. On the other hand, the fate mapping in related urodele *Pleurodeles waltl* shows a similar blastomeres contribution to the formation of body structures as in *X. laevis* (Delarue et al., 1997)*.* However, it is still unknown to what extent the gross similarities in blastomere fate-mapping reflect similarities in the distribution of molecular components along the A-V axis.

In our laboratory, we focus on the identification of asymmetrically distributed biomolecules in oocytes and early embryos of many animal species. Recently, we identified about 15000 maternal transcripts asymmetrically localized along the animal-vegetal axis in *X. laevis* oocytes (Sindelka et al., 2018). These mRNAs were classified into four localization profile groups: extremely animal, animal, vegetal and extremely vegetal. We identified most of the mRNAs in the animal hemisphere—94%. The extremely animal group contains 2.8% mRNAs, which are important mainly in transcription and translation regulation. While animal localization is probably formed through diffusion, the localization in extremely animal sections seems to be caused by the yet undiscovered active transport mechanism. In the vegetal and extremely vegetal sections, we identified 1.3% and 0.2% of the total mRNAs respectively.

Previous studies have shown the presence of three distinct pathways for vegetal RNA localization. The first one is called the early pathway (also known as METRO) and is used mainly for the localization of germ plasm determinants such as *nanos1* (Forristall et al., 1995; Kloc and Etkin, 1995; Zhou and King, 1996), *dazl* (Houston et al., 1998) and *ddx25* (MacArthur et al., 2000)*.* During early oogenesis, mRNAs diffuse from the nucleus to be entrapped by the mitochondrial cloud (in fish, called Balbiani body). Later the whole structure is transported towards the vegetal pole to be anchored in the narrow region of the oocyte vegetal cortex (Forristall et al., 1995; Chang et al., 2004). The localization through the late pathway takes place at later stages of oogenesis. This pathway includes mainly mRNAs essential for the germ layer development, such as *gdf1* (also called *Vg1*) (Melton, 1987; Forristall et al., 1995; Kloc and Etkin, 1995; Deshler et al., 1997) and *vegt* (Lustig et al., 1996; Stennard et al., 1996; Zhang and King, 1996). The late pathway components are localized to the vegetal region by a microtubule-dependent mechanism and then anchored in the wide region of the vegetal cortex. In addition, the existence of mRNAs sharing some characteristics of both major pathways led to the categorization of the new intermediate pathway. Examples of such mRNAs include *dnd1* (Horvay et al., 2006)*, grip2* (Clauβen et al., 2011) and *plin2* (Chan et al., 1999).

In anuran amphibians and teleost fish, the primordial germ cells (PGCs) are produced from germ plasm determinants that migrated to the vegetal hemisphere during oogenesis. (Mahowald and Hennen, 1971; Whitington and Dixon, 1975; Heasman et al., 1984; Knaut et al., 2000). This mechanism of PGCs formation, known as preformation, involves germ plasm repression of transcription of somatic genes in the primordial germ cells (PGCs) leading to germ line segregation (Venkatarama et al., 2010). Another mechanism of PGCs determination, epigenesis (also called induction), is found in *M. musculus*, and involves the production of PGCs through the induction of pluripotent cells of early gastrula by extracellular signals in a germ plasm-independent manner (Tam and Zhou, 1996). Germ plasm has never been observed in urodele oocytes or eggs and therefore it is believed that the germ line of these amphibians is also most probably determined by epigenesis (Johnson et al., 2001).

In teleost fish, such as *D. rerio*, the maternal determinant gradients along the animal-vegetal axis are established during oogenesis similarly to amphibians. Surprisingly, these gradients are disrupted shortly after fertilization in the RNA translocation phenomenon, which is indispensable for germline and germ layer development. This is observed for the vegetally localized germ plasm components (*dnd1, nanos1* and *ddx4*) which migrate to the animal pole after fertilization (Howley and Ho, 2000; Weidinger et al., 2003; Theusch et al., 2006). The post-fertilization translocation is connected with the cytoplasm segregation from the vitelloplasm resulting in the creation of a blastodisc, that will give rise to the embryo (reviewed in Fuentes et al., 2018). The process is accompanied with slow and fast cytoplasmic flow. While the actin-dependent slow cytoplasmic flow translocates vegetally localized *dazl* towards the animal pole, fast cytoplasmic flow transports dorsal determinants (*grip2a* and *wnt8a*) along microtubules to the dorsal side of the embryo (Lu et al., 2011; Tran et al., 2012; Ge et al., 2014; Welch and Pelegri, 2015). During cell division, the cells at the base of blastodisc containing germ plasm markers adopt a germ cell lineage fate (Hashimoto et al., 2004; Kosaka et al., 2007).

When the animal-vegetal axis is established the determination of the left-right and dorsal-ventral axis can start. The first step in the establishment of the dorsal-ventral axis occurs in *X. laevis* shortly after fertilization. The sperm penetrates to the future ventral side and this event leads to the process known as cortical rotation. The cytoplasmic movement and cytoskeleton reorganization give rise to a grey crescent, which is the base for the origin of the Niewkoop center and the gastrulation induction center—The Spemann organizer (Vincent et al., 1986; Vincent et al., 1987). The Spemann organizer is formed through the crosstalk of two signaling pathways (Agius et al., 2000; Nishita et al., 2000). For the activation of the Wnt pathway, it is necessary to stabilize β-catenin on the dorsal side of the embryo. The stabilizing factors are originally present in the vegetal hemisphere but are transported to the dorsal side after the cortical rotation. Here, stabilizing factors can act on β-catenin leading to the expression of zygotic genes (*siamois, twin*) (Brannon et al., 1997; Laurent et al., 1997). The second signaling pathway occurs shortly after fertilization. It is known that some vegetally localized mRNAs (*vegt*) are translated after fertilization and proteins diffuse to the equatorial region of the egg. These proteins regulate the gene expression of *transforming growth factor-beta* (TGFβ) family members (Kofron et al., 1999) The cooperation of β-catenin*-*activated genes and TGFβ family genes direct the formation of Spemann organizer and initiate gastrulation (Agius et al., 2000; Nishita et al., 2000).

While the role of asymmetric RNA distribution in the animal-vegetal axis establishment has been confirmed, the induction of dorsal-ventral and left-right axes appears to be independent on RNA localization in *X. laevis*. In (Levin et al.,2002) revealed the asymmetric distribution of mRNA H+-V-ATPase using *in situ* hybridization and he outlined a possible role in left-right axis induction, but this hypothesis has been disproven a few years later. The single blastomere transcriptome analysis of 8-cell stage *Xenopus tropicalis* embryos also revealed the absence of any RNA pattern along the dorsal-ventral and left-right axis (De Domenico et al., 2015). Moreover, in our laboratory, we analyzed the expression of genes that have been previously connected with the dorsal-ventral pattern (for example *dvl2, dvl3, gsk3b, ctnnb1* and *wnt11*) and confirmed the non-existence of RNA asymmetry (Flachsova et al., 2013). These results indicate the involvement of other biomolecules, such as proteins, in the establishment of these axes in anurans.

Previously, we compared localization profiles of matured eggs along the animal-vegetal axis among various model organisms (*X*. *laevis, D*. *rerio, A*. *mexicanum* and *Acipenser ruthenus*) and revealed the relatively low conservation in RNA localization (Naraine et al., 2022). Here, we continue using urodele *A*. *mexicanum* to study the spatiotemporal changes during early development in comparison to anuran amphibians. We identified asymmetrically localized RNAs along the animal-vegetal axis and revealed that many identified RNAs show dynamic pattern changes in stages before the onset of mid-blastula transition (MBT), the event of embryonic genome activation. The detected changes were classified into two groups. The first group contains genes that are transcribed *de novo* before MBT, showing the gradual activation of the embryonic genome. In the second group, there are genes whose transcripts are partially degraded after fertilization. Surprisingly many degraded genes are germ plasm markers suggesting preformation as a conserved mechanism for vertebrates as mentioned in (Škugor et al., 2016). Many of *de novo* or degraded transcripts shows altering profiles during development. In addition, we found motifs conserved in PGC transcripts of *A. mexicanum* and suggested its possible role in the early development of urodeles.

## Methods

### Ethics approval

All experimental procedures involving model organism *A. mexicanum* were carried out in accordance with the Czech Law 246/1992 on animal welfare. *A*. *mexicanum* animals were from the colony of the Department of Zoology, Faculty of Science, Charles University, Prague, Czech Republic, and all protocols were approved by the Faculty of Sciences of Charles University.

### Embryos collection

*A. mexicanum* male and female adults were kept together in an aquarium and after natural stimulation, the females laid eggs. Samples were prepared in two independent experiments and using two different females and males. The gel envelope was first removed from the eggs using tweezers. Eggs were then collected and incubated in sterile 1× Steinberg’s solution containing Pen-Strep (Sigma). Embryos at the 1-, 4-, 64- and 1K-cell stages were embedded in Tissue-Tek O.C.T. Compound (Sakura) with the animal pole oriented at the top. All samples were then stored in the freezer at −80°C.

### Sample preparation

**FIGURE 1:**
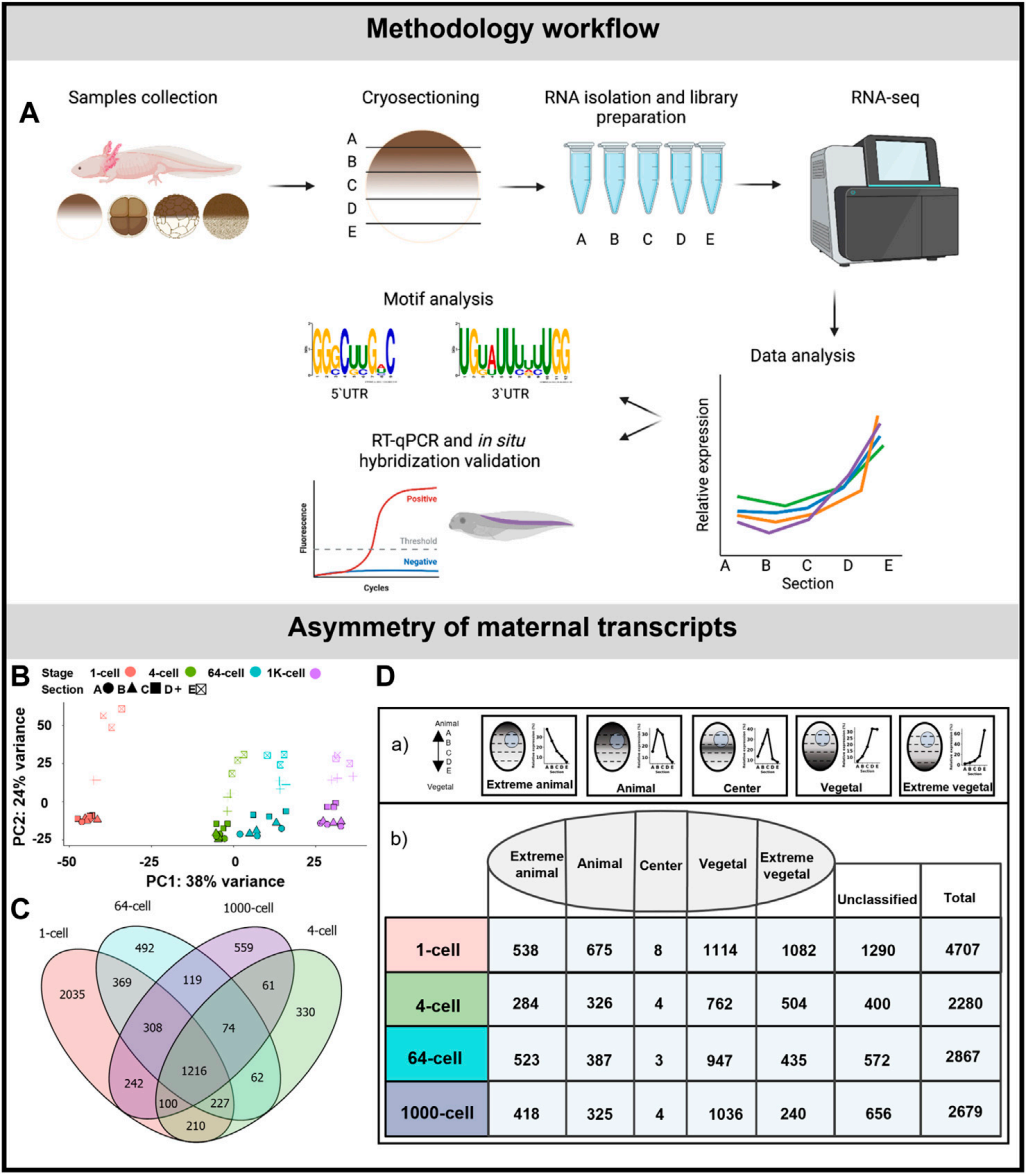
Asymmetric localization of maternal transcript in *A. mexicanum* early embryos. **(A)** Schematic representation of the workflow. **(B)** PCA of 500 most variable transcripts show high variability among developmental stages and embryo sections. **(C)** The diagram of shared DLTs among developmental stages. **(D)** Number of DLTs in each localization category. DLTs in the unclassified category represent those that did not fit into any of the five defined profiles.

Samples were subsequently incubated in the cryostat chamber (Leica CM 1950, USA) at −24°C for 10 min and then cut into 30 μm slices along the animal-vegetal axis as shown in Figure 1A. The slices were pooled and equally distributed into 5 tubes. The tube labelling corresponded to embryo orientation: A—extremely animal segment, B—animal, C—central, D—vegetal, E—extremely vegetal.

### RNA isolation and reverse transcription

The samples were homogenized in 300 μl of TRIReagent^®^ (Sigma-Aldrich, USA) and total RNA was extracted according to the manufacturer’s protocol. LiCl precipitation was performed to remove inhibitors present in the yolky vegetal hemisphere. The concentration of RNA was measured using NanoDrop-2000 (ThermoFisher, USA) and sample quality was assessed using 5200 Fragment Analyzer (Agilent, USA).

The cDNA was prepared using 30 ng of total RNA and RNase-free distilled water (ThermoFisher, USA) in a volume of 5.5 μl and a reaction mixture was added containing 0.5 μl of dNTPs (10 μM each, ThermoFisher, USA), 0.5 μl of oligo-dT and random hexamer (1:1 mixture, 50 μM each, ThermoFisher, USA), and 0.5 μl of RNA-spike (TATAA biocentre, Sweden). The mixture was incubated for 5 min at 65°C and 10 min at 4°C. During the second step, the second mixture was added containing 2 μl of 5xRT Buffer (ThermoFisher, USA), 0.5 μl of RnaseOUT (ThermoFisher, USA) and 0.5 μl of Maxima H Minus Reverse Transcriptase (ThermoFisher, USA). The reaction proceeded as follows—10 min at 25°C, 30 min at 50°C, 5 min at 85°C and cooling to 4°C. The cDNA was diluted to 100 μl using Tris-EDTA buffer solution (Sigma-Aldrich, USA) and stored at −20°C.

### Primer design and qPCR

qPCR was performed to detect the localization of known genes (list of used primers is attached in Supplementary Table S1). PCR primers were designed using Primer3 (Untergasser et al., 2012). The expected length of qPCR products was 80–120 bp and the annealing temperature was 60°C. Geneious prime (version 2021.2) was used to increase the specificity of designed primers and to avoid targeting RNA isoforms.

qPCR reaction mix with a total volume of 7 μl contained 2 μl of cDNA, 0.29 μl of forward and reverse primers mix (1:1, 10 μM each), 3.5 μl of 2x TATAA SYBR^®^ GrandMaster^®^ Mix (TATAA Biocenter, Sweden) and 1.21 μl of RNase-free distilled water (ThermoFisher, USA). qPCR was performed using CFX384 Real-Time System (Bio-Rad, USA) as follows: the initial denaturation for 3 min at 95°C, 45 cycles of denaturation for 15 s at 95°C, annealing at 60°C for 20 s and extension at 72°C for 20 s. qPCR melting curves were analyzed to test the reaction specificity. qPCR data were analyzed using workflow published in Sindelka et al., 2008.

### Library preparation

We used 100 ng of a total RNA for library preparation. Ribosomal RNA depletion was performed using Ribocop rRNA Depletion Kit V1.3 (Lexogen, Austria). Libraries were prepared using NEBNext^®^ Ultra™ II Directional RNA Library Prep Kit for Illumina^®^ (New England Biolabs, USA). The number of PCR cycles was set at 12 cycles according to the initial RNA concentration. Library concentration was measured with a Qubit 4 Fluorometer (ThermoFisher, USA) and quality was assessed using a 5200 Fragment Analyzer (Agilent, USA). The pooled libraries were sequenced using Illumina NextSeq 500, high-output 150 bp run.

### Molecular cloning

The cDNA of *grip2, dnd1, rbpms2* and AMEXTC_0340000004005 was PCR amplified using primers (Supplementary Table S2) designed for a full length cDNA. PCR reaction mix contained 5x Phusion green HF buffer (ThermoFisher, USA), 10 μM MgCl_2_ (ThermoFisher, USA), 10 μM dNTP_3_ (ThermoFisher, USA), Phusion Hot Start II DNA Polymerase (ThermoFisher, USA), UltraPure dH_2_O (Invitrogen, USA) and forward and reverse primers. PCR program run as follows: initial denaturation at 98°C for 30 s, 39 cycles of denaturation for 10 s, annealing at 55°C for 30 s and extension at 72°C for 2 min.

The plasmid pBluescript II KS+ and amplified cDNA were digested using *XhoI* and *NotI* (New England Biolabs, USA). 5′-ends of DNA were dephosphorylated using CIP (calf intestinal alkaline phosphatase, New England Biolabs, USA) and then DNA insert was inserted into vector DNA in a ligation reaction. Ligation mix contained T4 DNA Ligase Buffer (New England Biolabs, USA), vector DNA, insert DNA, RNase-free distilled water (ThermoFisher) and T4 DNA ligase (New England Biolabs, USA). The mix was incubated overnight at 16°C. Then, the reaction was stopped at 65°C for 10 min.

NEB 5-alpha competent *E. coli* was used for a transformation (High efficiency, # C2987I, New England Biolabs, USA) according to the manufacturer’s protocol: High Efficiency Transformation Protocol (C2987H/C2987I). After that, the individual clones were sequenced to screen for the presence of the expected sequences. Correct plasmids were purified using Plasmid Midi Kit (Qiagen, Germany).

### Probes preparation and whole mount *in situ* hybridization

Plasmids were linearized in the restriction digest reaction. The mix contained 7 μg of plasmid DNA, NEB restriction enzyme (New England Biolabs, USA) and 10x NEB buffer 3.1 (New England Biolabs, USA). Mix was incubated overnight in a 37°C water bath. Linear DNA was cleaned up using QIAquick PCR Purification Kit (Qiagen, Germany). During the transcription reaction, we mixed 2 μg of linear template, 4 μl of 5x transcription buffer (Agilent, USA), 2 μl DIG RNA labelling mix (Roche, Switzerland), 2 μl of the T7 polymerase (Agilent, USA) and water in total volume of 20 μl. The mixture was incubated in a 37°C water bath for 3 h. Then, the mix was cleaned using LiCl. Finally, the RNA probe quality was tested using formaldehyde gel.

*In situ* hybridization was performed on whole mounts as described previously (Soukup et al., 2021) with slight modifications. Briefly, rehydrated *A. mexicanum* albino (d/d) embryos were digested in 60 μg/ml Proteinase K in PBS, fixed in 4% formaldehyde + 0.2% glutaraldehyde for 30–120 min, transferred into hybridization solution (50% formamide, 1x Denhardt’s, 1 mg/ml yeast RNA, 0.1% Tween-20, 10% dextran sulfate, 1x salt solution containing 0.2 M NaCl, 8.9 mM Tris-HCl, 1.1 mM Tris base, 5 mM NaH_2_PO_4_.H_2_O, 5 mM Na_2_HPO_4_ and 5 mM EDTA), and incubated overnight in hybridization solution containing RNA probe (1:1,000–1:100). Next day, the specimens were washed several times in post-hybridization solution (50% formamide, 4 × SSC, 0.5% Tween-20) and transferred via MABT buffer (100 mM maleic acid, 150 mM NaCl, 0.1% Tween-20) into blocking solution (2% blocking reagent, 20% sheep serum, in MABT buffer). Following blocking, the specimens were incubated overnight in the blocking solution containing alkaline phosphatase-conjugated antibody against DIG (Roche, 1:3,000) at 4°C. The specimens were washed several times in the MABT buffer. Following the overnight MABT wash, the samples were transferred into NTMT buffer (0.1 M Tris, 0.1 M NaCl, 0.05 M MgCl_2_, 0.1% Tween-20) and incubated in BM Purple substrate (Roche) at 4°C until desired signal developed.

### RNA-seq data processing and analysis

RNA-seq reads were processed as previously described in Naraine et al. (2022). Adaptor sequences and low quality reads were removed using TrimmomaticPE (v. 0.36) (Bolger et al., 2014) using the parameters, “HEADCROP:12 ILLUMINACLIP:∼/TruSeq-PE3.fa:2:30:10 LEADING:3 TRAILING:3 SLIDINGWINDOW:4:15 MINLEN:36.” Mitochondrial RNA reads (GenBank id: AY659991.1) and any remaining rRNA reads were removed using SortMeRNA (v. 2.1b) (Kopylova et al., 2012). The reads were then pseudo-aligned to the *A. mexicanum* transcriptome AmexT_v34 (Nowoshilow and Tanaka, 2020) using kallisto (v. 0.43.1) (Bray et al., 2016). The data were deposited in the National Center for Biotechnology Information’s Gene Expression Omnibus (GEO: GSE240796).

Raw counts were initially filtered to keep transcripts with counts greater than 30 in at least one sample. DESeq2 (v. 1.32.0) (Love et al., 2014) was used to normalize the counts using the median-of-ratios method followed by differential expression analysis to determine differential localization of transcripts along the animal-vegetal sections in the 1-cell, 4-cell, 64-cell, 1K-cell stages; changes in the sectional profile across all stages; and changes in the total transcript across the stages. The median-of-ratios normalization method was used to focus primarily on the extremely localized transcripts.

The following DESeq2 design models were used:

1) Alteration between the sections at the same stage:
  a) design: ∼replicate + position; reduced design: ∼replicate
2) Alteration in the profiles across the different stages:
  a) Transcripts with altered profiles: design: ∼Stage + position + Stage:position; reduced: ∼Stage + position
  b) Transcripts with altered magnitudes: design: ∼Stage + position; reduced: ∼Stage
3) Alteration of the total transcript count between the different stages:
  a) design: ∼Stage; reduced: ∼1; uses the sum of the normalized counts for each sample as input counts

The Principal Component Analysis (PCA) of the top 500 variable transcripts was assessed for the presence of any outlier samples. Differentially localized transcripts (DLTs) were defined as those with an adjusted *p*-value (padj) value less than 0.01 and also a total transcript count greater than 20 within at least one stage. Human gene symbols were assigned based on the previous ortholog analysis from Naraine et al., 2022, whereby *A. mexicanum* gene symbols were either matched against all known *Homo sapiens* gene symbols or derived from the similarity between its protein sequences as compared against the *H. sapiens* proteome using the reciprocal best alignment heuristic tool Proteinortho (v. 6.0.9) (Lechner et al., 2011).

The spatial expression profiles of the DLTs were then characterized into five discrete categories (extreme animal, animal, central, vegetal, extreme vegetal) based on the parameters previously described in Naraine et al., 2022. The central category represents a new category of localized maternal transcripts that was observed primarily in the eggs of *A. mexicanum* and *A. ruthenus* (Naraine et al., 2022). The other four profile parameters remained unchanged from the previous publication describing sub-location in *X.* laevis (Sindelka et al., 2018). DLTs that did not fit into these defined parameters were labelled as unclassified. Profile changes in the DLTs across the whole stages and within the stages were assessed using the degPatterns function from DEGreport (v. 1.28.0) package (Pantano et al., 2023). Profiles where the DLTs showed a fold change of 3x or 2x difference between either the stages or the sections respectively were selected. The validity of the profiles was verified using optCluster (v. 1.3.0) with the “Diana” clustering algorithm (Sekula et al., 2017).

Gene ontology terms associated with the genes were obtained using online software g:Profiler (access date: 14/02/23) using the default parameters of the annotated human reference, multiple testing correction using g:SCS threshold with a cutoff of 0.05 (Raudvere et al., 2019). Gene ontology terms clustering and removal of redundant terms were done using Revigo (access date: 14/02/23) with the default parameters of the whole UniProt reference database and SimRel semantic similarity measurement (Supek et al., 2011).

### Motif analysis

We analyzed the presence of motifs unique within the UTRs of *A. mexicanum* PGC markers that might be responsible for its degradation. This was done by comparing their 3′ and 5′UTRs against shuffled versions of their sequences or the UTRs from *X. laevis* PGC genes. We also analyzed for enrichment of motifs within the vegetal and animal transcripts using the previously observed vegetal and animal motifs that were detected in *A. mexicanum* oocytes (previously published by Naraine et al., 2022). Analyzing the 3′UTR sequences, we also checked for *de novo* motifs that might be enriched selectively within the degraded transcripts versus the *de novo* transcripts and also between the animal and vegetal groups.

Motifs were detected using the STREME software (v. 5.5.2) (Bailey, 2021) under the following conditions: *p*-value < 0.05 and motif width = 6 to 25. The motif enrichment in primary sequences compared with control sequences was assessed using AME (e-value ≤ 0.05) (v. 5.5.2) (McLeay and Bailey, 2010). FIMO software (*p*-value ≤ 0.0001) (v. 5.5.2) (Grant et al., 2011) was used to scan the identified motifs against primary and control sequences. Using FIMO we obtained information about motif position within the sequences, and it was used to assemble a map of the motif distribution.

The enrichment of the previously observed vegetal and animal motifs from the paper (Naraine et al., 2022) was analysed against the UTRs of the animal and vegetal transcripts observed during embryogenesis. Significantly enriched motifs were deemed as those that gave an AME e-value ≤ 0.05 and a 3x fold enrichment in the vegetal UTRs versus the animal UTRs. In the case of the occurrence of the same or very similar motifs, we selected the motif with the lowest e-value.

Using only the PGC dataset, we continued with the analysis of putative regulatory elements in the UTRs that might explain its temporal degradation. To identify known protein binding sites and RNA binding proteins (RBPs) within the 3′UTR, we used Scan For Motif (access date: 16/03/23) with the datasets from TransTerm (E-value <= 0.175 per thousand bases) and RBPDB (E-value <= 0.001) and selected results with E-value thresholds < 0.001 (Biswas and Brown, 2014). The motifs recognized by these RBPs were compared against the list of *de novo* motifs detected by STREME using the comparison tool Tomtom (v. 5.5.2) (Gupta et al., 2007). The position probability matrices for the motif of these RBPs were downloaded from several online databases: CIS-BP-RNA (v. 0.6) (Ray et al., 2013), RBPDB (v. 1.3.1) (Cook et al., 2011) and oRNAment (access date: 12/06/23) (Benoit Bouvrette et al., 2020). Within 3′ and 5′UTR, the BEAM software (v. 1.6.1.) (Pietrosanto et al., 2018) was used to find RNA secondary structures using a *p*-value threshold cutoff of 0.01. The BEAGLE software (access date: 23/05/23) (Mattei et al., 2015) was then used to identify a conserved secondary structure either shared across all the *A. mexicanum* PGCs or unique to *A. mexicanum* relative to the PGCs in *X. laevis*, using a *p*-value threshold cutoff of 0.01. BRIO (*p*-value < 0.05) (Guarracino et al., 2021) was used to identify known sequences and structure RNA-binding motifs that are recognized by RBPs in the UTRs of *H. sapiens* and *M. musculus* from PAR-CLIP, eCLIP and HITS experiments. The AURA database (v. 2.7) was then used to screen the identified RBPs for their selective preference to either the 3′ or 5′ UTR regions (Dassi et al., 2014). For the discovery of putative binding sites for miRNA within the 3′UTR of the PGC markers, miRDB (v. 6.0) (Chen and Wang, 2020) was used with the human dataset as a reference.

## Results

### Asymmetric distribution of maternal RNA in early embryos

To describe RNA localization during early embryogenesis of *A. mexicanum*, we collected the stage of a fertilized egg (1-cell stage), 4-, 64- and 1K-cell (early blastula), which are expected to be pre-embryonic genome activation (MBT).

Embryos were sectioned along the animal-vegetal axis using the TOMO-seq method (Figure 1A). The results were first analyzed using PCA of the 500 most variable transcripts (Figure 1B). PC1 showed high variability during early development, mainly between the 1-cell stage and late embryos. PC2 revealed the clear distinction among individual sections in all stages. The variability in the sections decreased as the embryos progressed towards the later stages, indicating a disruption or reduction in the original asymmetrical gradients. Additionally the largest sectional differences were observed between the D and E sections, while minimal between the A and B sections.

The average number of coding genes identified at each stage was 28909 (using threshold >30 transcript counts in any sample). The complete dataset containing genes with asymmetrical distribution are listed in Supplementary Table S3. More than 2,200 DLTs (padj < 0.01, using threshold >20 transcripts in at least one stage) with sectional changes per stage were identified in embryos at 4, 64 and 1K-cell stages and twice as many were identified at the 1-cell stage (4,076 DLTs). The diagram in Figure 1C shows the overlap of the shared DLTs across the stages with 1,216 shared in all analyzed stages. Most of the DLTs were classified into one of the five localization categories: extremely animal, animal, central, vegetal and extremely vegetal (Fig. 1Da). In extremely animal and animal categories, we identified 284–675 DLTs in each stage (Fig. 1Db). Majority of DLTs are localized in vegetal or extremely vegetal sections at the 1-cell stage—1,114 and 1,083. The number of extreme vegetal DLTs dramatically decreased during development and only 240 were found at the 1K-cell stage.

We performed RT-qPCR validation of a few members within the extremely vegetal and animal transcript categories. In the extremely vegetal category, we confirmed the localization profile of *grip2* and *dnd1* (Supplementary Figure S1A), and in the animal category, we confirmed the localization of *ankhd1* and *akt2* (Supplementary Figure S1B).

### Sectional profile alteration during the early development

In total 4,850 DLTs were observed to change their sectional profile or its sectional amplitude across the analyzed stages. 781 of these DLTs had a ≥2x fold change between a given section and showed two profile alteration processes—alteration of vegetal profiles and alteration of animal profiles (Supplementary Table S4).

#### Vegetal alteration

**FIGURE 2:**
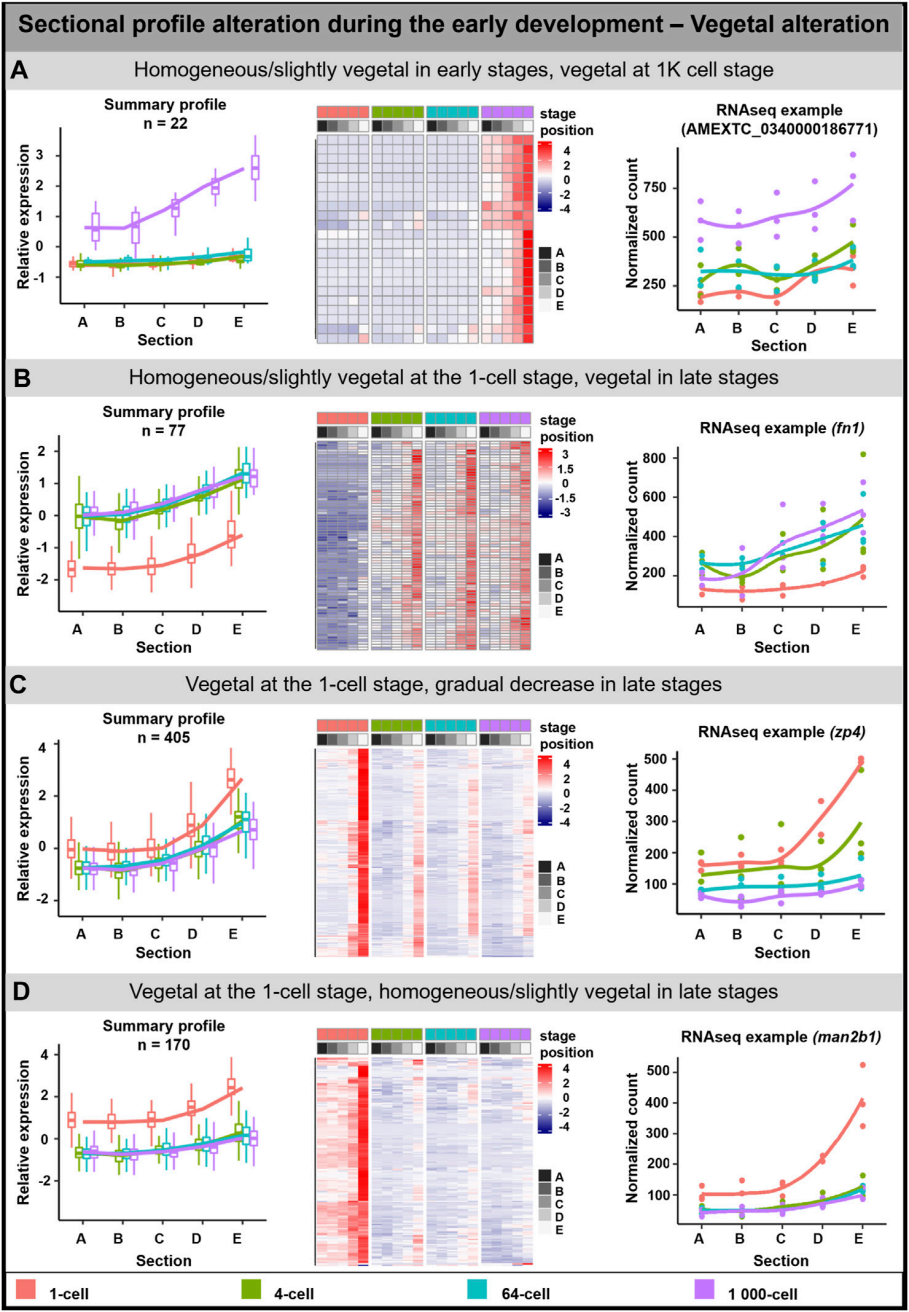
Vegetal sectional profile alteration. During the early development of *A. mexicanum,* 4 groups of vegetal DLTs altering profiles were observable. **(A)** Homogenous or slightly vegetal localization from the 1-cell stage until the 64-cell stage and vegetal localization at the 1K-cell stage. **(B)** Homogenous or slightly vegetal localization at the 1-cell stage and vegetal localization from the 4-cell stage and later. **(C)** Vegetal localization at the 1-cell stage and from the 4-cell stage until the 1K-cell stage the gradual decrease of transcript amount. **(D)** Vegetal localization at the 1-cell stage and from the 4-cell stage the localization is homogenous or slightly vegetal. Line plots represent the averaged z-score expression for the genes with shared localization profiles. Heatmap shows the z-score of the averaged relative expression across the replicates. DLTs represent genes that had a padj < 0.01 and greater than 20 transcripts per stage. DLTs were further filtered to show those that were 2x greater in either amplitude or relative to another section across the stages. 3 biological replicates were used. Embryos sections: A - extremely animal, B - animal, C - central, D - vegetal, E - extremely vegetal.

The vegetal alteration was observed for 673 DLTs and several groups depicting different types of alterations were described (Figure 2). A pronounced vegetal profile was created either at the 1K cell stage (22 DLTs, Figure 2A) or from the 4-cell stage (77 DLTs, Figure 2B). Next group showed the vegetal profile already established at the 1-cell stage (405 DLTs), while a uniformed/reduced distribution during the other developmental stages (Figure 2C). In the last group, the vegetal profile is also present at the 1-cell stage (Figure 2D). However, in contrast to the slow progression towards the uniform distribution, it was already established at the 4-cell stage (169 DLTs).

Due to the low number of genes in the mentioned groups or the limited number of annotated genes, the GO enrichment analysis was performed only on the third group (Vegetal at the 1 cell/Gradual decrease in late stages; Supplementary Table S5). Enriched GO terms were associated with biological processes affiliated with localization and protein folding, and molecular function in oxidoreductase activity and protein binding.

#### Animal alteration

**FIGURE 3:**
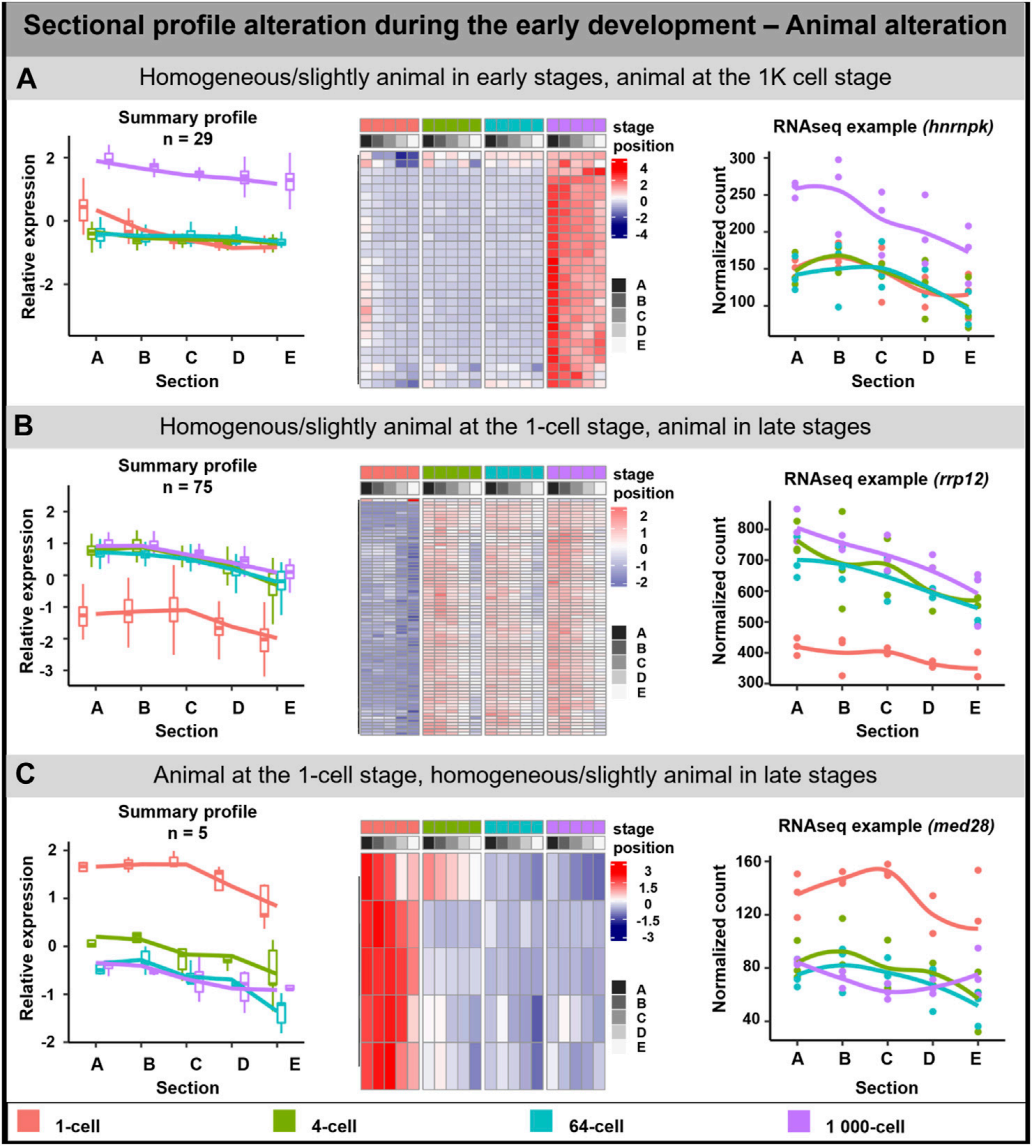
Animal sectional profile alteration. During the early development of *A. mexicanum,* 3 groups of animal DLTs altering profiles were observable. **(A)** Homogeneous or slightly animal from 1-cell until the 64-cell stage and the creation of animal profile at the 1K-cell stage. **(B)** Homogeneous or slightly animal at the 1-cell stage and animal at the 4-cell stage and later. **(C)** Animal localization at the 1-cell stage and homogenous or slightly animal localization from 4-cell until the 1K-cell stage. Line plots represent the averaged z-score expression for the genes with shared localization profiles. Heatmap shows the z-score of the averaged relative expression across the replicates. DLTs represent genes that had a padj < 0.01 and greater than 20 transcripts per stage. DLTs were further filtered to show those that were 2x greater in either amplitude or relative to another section across the stages. 3 biological replicates were used. Embryos sections: A – extremely animal, B – animal, C – central, D – vegetal, E – extremely vegetal.

The animal profile alteration comprised of 108 DLTs which can be classified into three groups (Figure 3). In the first group, the transcript uniform distribution persisted until the stage of 64-cell, after which at 1K-cell the animal profile was more visible (29 DLTs, Figure 3A). DLTs in the second group were uniformly distributed only at the 1-cell stage and later formed animal gradients (74 DLTs, Figure 3B). The third group showed clear animal profile at the 1-cell (5 DLTs, Figure 3C), but its animal distribution was disrupted leading to homogenous distribution in the later stages.

GO enrichment was performed only on the second group (Homogenous/slightly animal at the 1-cell stage, animal in late stages) as it contained sufficient numbers of genes. The genes in this group may play a role as the cellular components of the centrosome, cytoplasm, and cytoskeleton. No molecular function or biological process connected with the transcripts in this group was observed.

### Transcript count alteration during the early development

Alteration in transcript count caused by synthesis or degradation was detected for 6,811 DLTs (padj <0.01, >20 transcripts in at least one stage). Out of these, we observed 960 DLTs with at least 3-fold change across individual stages (Supplementary Table S6). Two main groups were created reflecting *de novo* synthesis and degradation.

Transcripts degradation detected using the TOMO-seq approach was validated by RT-qPCR for 2 DLTs—*plin2* and *velo1* (Supplementary Figure S1C)*. De novo* synthesis of *mok* and *nynrin-like* was also validated by RT-qPCR (Supplementary Figure S1D).

#### *De novo* transcription in early embryogenesis

**FIGURE 4:**
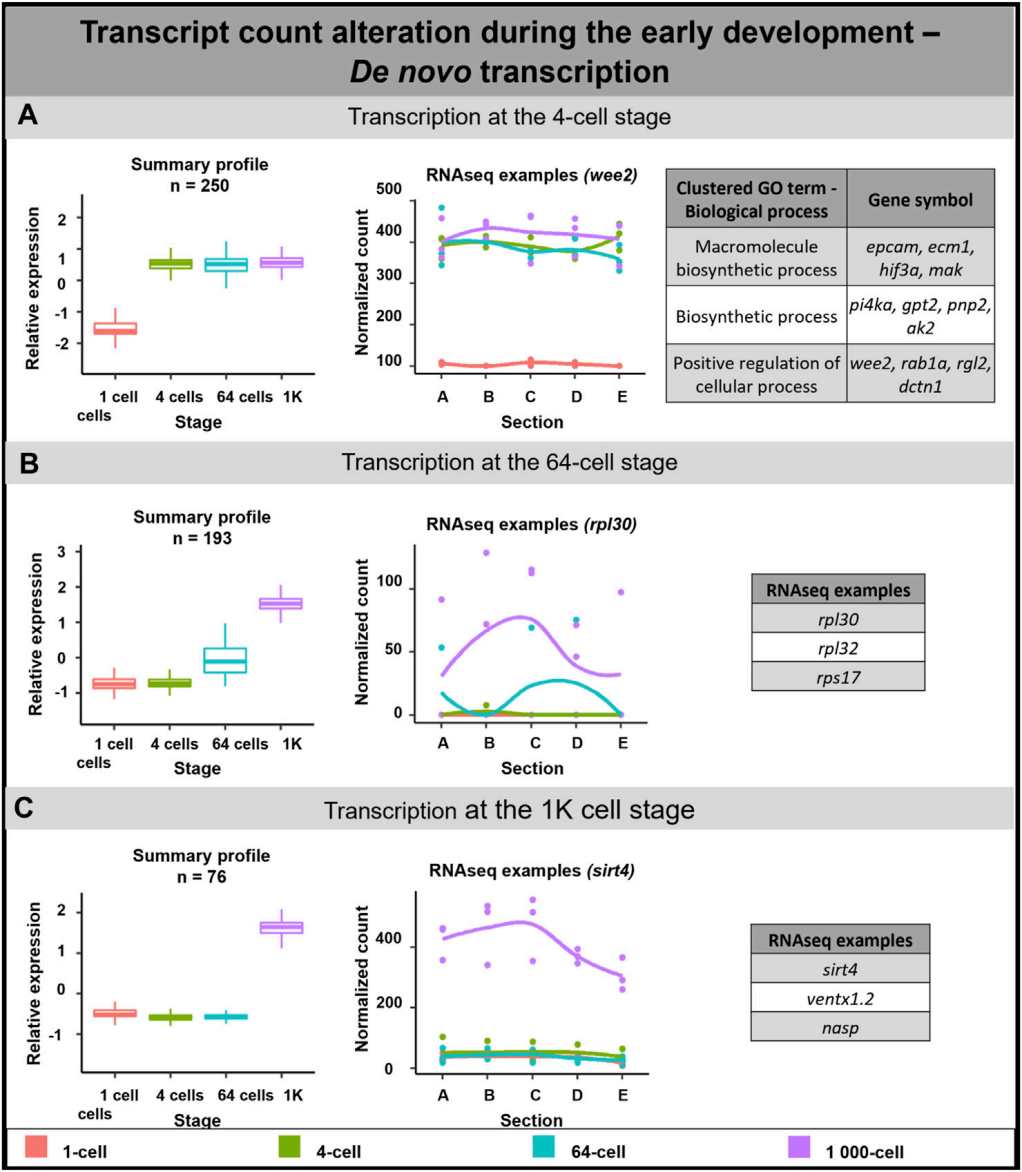
*De novo* transcription during early embryogenesis of *A*. *mexicanum*. **(A)** *De novo* transcription at the 4-cell stage and biological role of DLTs proposed using gene ontology analysis. **(B)** *De novo* transcription at the 64-cell stage and RNA-seq gene examples. **(C)** *De novo* transcription at the 1K-cell and RNA-seq gene examples. Box plots in the first column represent the averaged z-score expression for the averaged total transcript across the stage replicates. Line plots in the second column represent the normalized counts for each replicate for a specific gene across the stages. DLTs represent genes that had a padj < 0.01 and greater than 20 transcripts per stage. DLTs were further filtered to show those that were 3x greater in either amplitude or relative to another section across the stages. 3 biological replicates were used. Embryos sections: A - extremely animal, B - animal, C - central, D - vegetal, E - extremely vegetal.

*De novo* transcription of 519 DLTs was detected in the analyzed time points (Figure 4). 250 DLTs showed transcript increase right after fertilization at the 4-cell stage (Figure 4A). Out of these DLTs, 19 established vegetal profiles during development (e.g., *dusp1*) and 14 created animal profiles after the 1-cell stage (e.g., *prmt1*) (Supplementary Table S7).

The second wave of *de novo* transcription was observed at the 64-cell stage (193 DLTs, Figure 4B). Only 3 DLTs set up a vegetal profile at the 1K cell stage and 1 DLT created an animal profile at the 1K cell stage (not annotated transcripts).

The third *de novo* transcription was detected at the 1K-cell stage with 76 DLTs being synthesized at this time point. 6 DLTs showed preferential enrichment to the animal section (e.g., *rpl12*) and only 1 DLT was synthesized in the vegetal hemisphere at the 1K-cell stage (not annotated transcript).

Gene ontology terms associated with transcripts synthesized at the 4-cell stage proposed their role mainly in biological processes such as cell regulation and biosynthesis. Although it was not possible to perform gene ontology analysis on the remaining subgroups due to missing gene annotations, several interesting genes were revealed. For example, we identified several DLTs linked with ribosomal proteins at the 64-cell stage.

#### Transcript degradation during early embryogenesis

**FIGURE 5:**
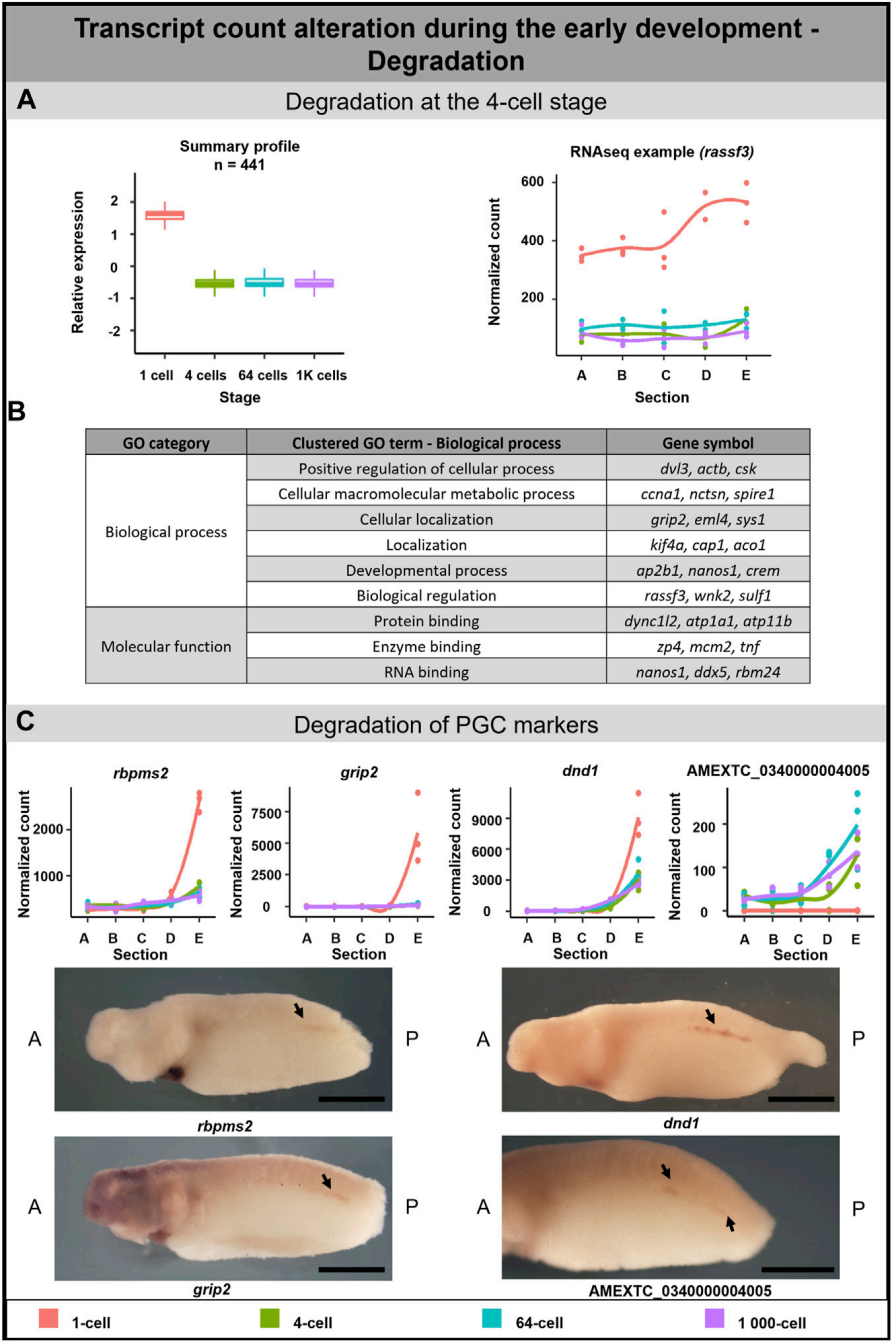
Transcription degradation during early embryogenesis of *A. mexicanum*. **(A)** The massive degradation was observed only after the 1-cell stage. Box plot in the first column represents the averaged z-score expression for the averaged total transcript across the stage replicates. Line plot in the second column represents the normalized counts for each replicate for a specific gene across the stages. DLTs represent genes that had a padj < 0.01 and greater than 20 transcripts per stage. DLTs were further filtered to show those that were 3x greater in either amplitude or relative to another section across the stages. 3 biological replicates were used. **(B)** Gene ontology analysis of degraded DLTs. **(C)** Degradation of PGC markers. Line plots show the localization profile and total amount of selected PGC markers change during the development. Whole-mount *in situ* hybridization shows the gene expression of 3 known PGC markers and 1 unknown gene within presumptive germ cells (arrow). Detection of PGC in *A. mexicanum* embryos at around stage 33 using *in situ* hybridization. Lateral view, with A-Anterior, B-Posterior. Scale bar = 2 mm.

A 3-fold decrease in 441 DLTs was observed between the 1-cell and 4-cell stages (Figure 5A). Out of these, 2 DLTs initially localized in the animal hemisphere but later lost their localization pattern due to degradation after the 1-cell stage (not annotated transcripts). 28 DLTs (e.g., *sys1*) were vegetal only at the 1-cell stage and their profile became uniform at the 4-cell stage. 33 DLTs (e.g., *sh3bp4*) were gradually degraded after the 1-cell stage and kept a slight vegetal profile even at later stages (Supplementary Table S7). In contrast, no significant degradation cluster was detected at the 64 or 1K-cell stages.

Gene ontology analysis supported the role of degraded DLTs in many biological processes, such as cell regulation, localization and development (Figure 5B). Moreover, the GO enrichment analysis suggests a molecular function of DLTs in the binding of specific molecules, such as proteins or RNAs (Supplementary Table S5).

#### Degradation of PGC markers during *A. mexicanum* development

A separate subgroup included genes that are known as the PGC markers. In this group, we observed the degradation of DLTs affecting profile pattern—from extremely vegetal to slightly vegetal or homogeneous distribution (Figure 5C). The transcript level of all PGC transcripts—*dnd1, rbmps2-1, rbpms2-2, grip2-1, grip2-2, nanos1, velo1-1* and *velo1-2*—significantly decreased after the 1-cell stage (Figure 5C; Supplementary Figure S2A). Even if some transcripts did not meet the criteria to be included in the total transcript alteration (3-fold change) or sectional profile alteration (2-fold change), the profile change was pronounced enough to suggest that vegetal degradation is occurring. To identify whether degraded transcripts are present in PGC in later development, we selected 3 genes from this group showing vegetal degradation (*rbmps2-1*, *grip2-1, dnd1*) and 1 gene (AMEXTC_0340000004005) with the opposite trend—zero count at the 1-cell stage and *de novo* transcription mainly in the vegetal hemisphere from the 4-cell stage—for *in situ* hybridization. All transcripts were detected in the embryo at around stage 33 in the presumptive germ line (Figure 5C) and surprisingly also in the heart and pronephros (Supplementary Figure S2B).

### Motif enrichment in primordial germ cell markers

For a deeper understanding of the PGC markers degradation process, we performed motif analysis searching for any conserved regulatory sequence within the 3′ and 5’ UTR sequences. Using data from previous publication (Naraine et al., 2022) and Xenbase.org we obtained UTRs of 5 transcripts in each organism (Listed in Supplementary Figure S2A). There were no statistically significant motifs enriched within the PGCs RNAs of the *A. mexicanum* versus those from the *X. laevis*. However, this is most likely due to the low number of genes used for the analysis (∼5 genes). Due to this limitation, FIMO was instead used to assess whether the detected motifs were found exclusively or in a high proportion within the UTRs of the *A. mexicanum* PGCs versus the other models.

**FIGURE 6:**
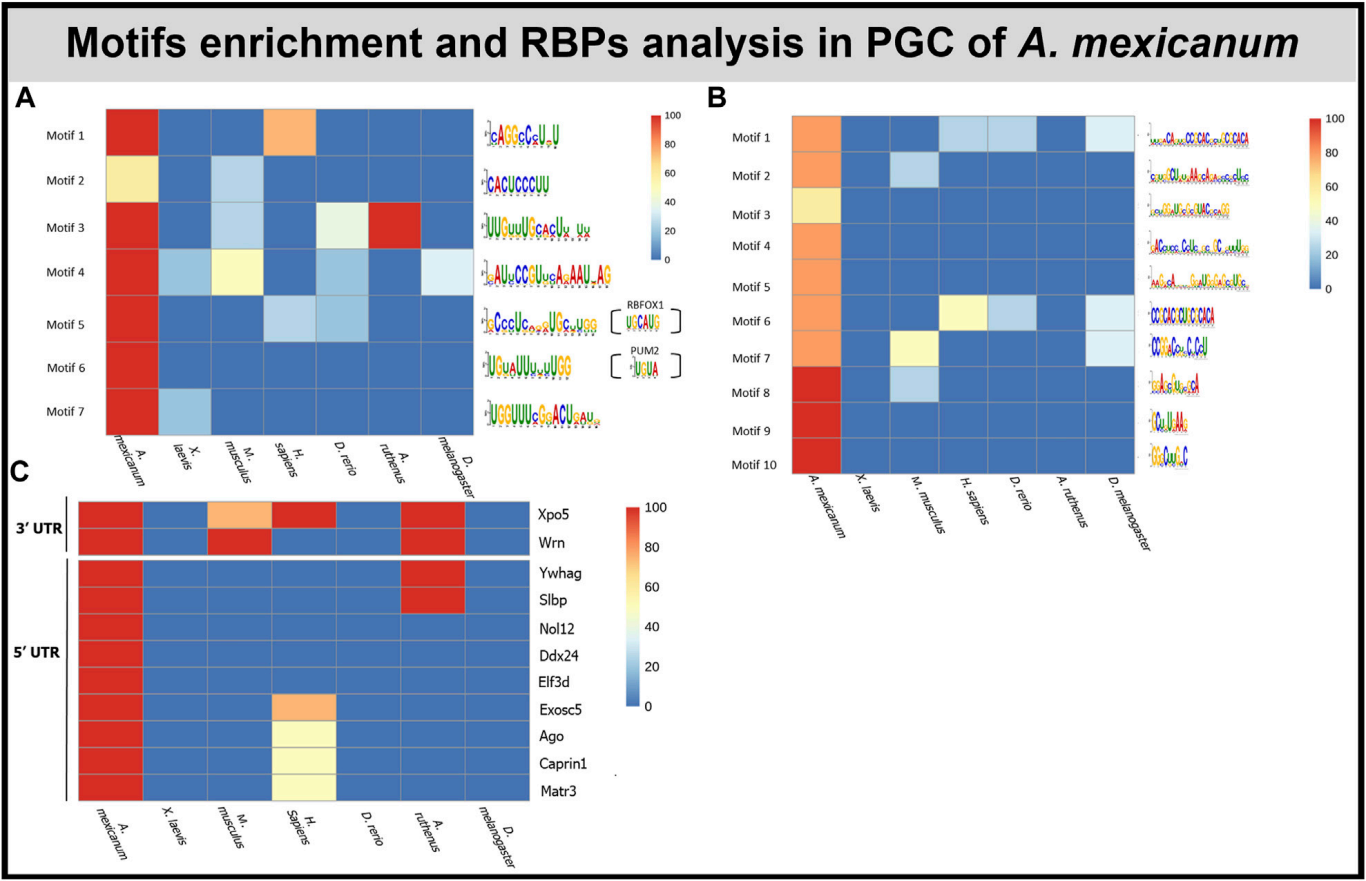
Motif and RBP enrichment in PGC. **(A)** *De novo* motif analysis within 3′UTR of *A. mexicanum.* Enriched motifs were also scanned in other model organisms. Sequences of motifs 5 and 6 probably can be bound by 2 known RBPS—PUM2 and RBFOX1. **(B)** *De novo* motifs analysis within 5′UTR of *A. mexicanum*. Enriched motifs were also scanned in other model organisms. **(C)** Identification of RBP binding motifs using BRIO within 3′ and 5′ UTR of *A.mexicanum*. The RBP presence was also assessed in other model organisms.

The *de novo* motif analysis within the 3′UTR of PGC marker genes revealed 7 motifs enriched in the *A. mexicanum* compared with either the *X. laevis* marker genes or the shuffled sequences (Figure 6A). Most of the motifs were uridine rich. 5 of the identified motifs were present exclusively in *A. mexicanum* but not in *X. laevis*. Then, the motif presence was also estimated in the other model organisms—*M. musculus, H. sapiens, D. melanogaster, A. ruthenus* and *D. rerio—*and only 1 motif (Motif 6) was exclusive to *A. mexicanum*. We observed no significant enrichment of these 7 motifs when comparing the *de novo* transcripts against the degraded ones, or the vegetal sectional changes against the animal sectional changes.

In total, we identified 10 motifs conserved within the 5′UTR of PGC markers in *A. mexicanum* and none of them were present in *X. laevis* or the shuffled sequences (Figure 6B). Most of the motifs were enriched with either cytosine or guanosine. Two motifs (Motif 1 and 6)—had CAC core. All the motifs were scanned against PGC sequences of the 4 model organisms and 4 motifs were unique exclusively to *A. mexicanum*. We observed no significant enrichment of these 10 motifs when comparing the *de novo* transcripts against the degraded ones, or the vegetal sectional changes against the animal sectional changes.

We used the Scan for motif software to identify putative RBP sites within the 3′UTR of PGC markers. The analysis revealed several RBPs which can affect RNA stability, degradation and translation, such as PUM2, KHSRP and ZFP36. However, all the RBPs affecting RNA stability were also detected in *X. laevis* PGC markers (Complete list of RBPs in Supplementary Table S8)*.* To compare *de novo* motifs with motifs recognized by known RBPs we used the Tomtom tool, which helped us to identify 2 motifs which resembled previously discovered motifs. Motif 5 was similar to RBFOX1 (UGCAUG) binding sites and motif 6 was similar to PUM2 (UGUA) and ZFP36 (UUAUUUAWK) (Figure 6A).

BRIO software was used to search for known primary sequences and secondary structures associated with RBP binding motifs within the 3′ and 5′ UTRs of *A. mexicanum* PGC markers. We identified 5 (3′UTR) and 17 (5′UTR) RBPs binding motifs unique to *A. mexicanum* compared with *X. laevis* (Supplementary Table S9). Using the available resources, we selected 2 (3′UTR) and 9 (5′UTR) proteins potentially playing a role in transcript degradation (Figure 6C). BEAM was used to find structural motifs present in the RNA secondary structure, but no motif was shared in all 5 *A. mexicanum* PGC markers within 3′ or 5′UTR.

As it is known, miRNA can induce mRNA degradation or translational repression (reviewed in O’Brien et al., 2018). Therefore, we searched for miRNAs that may target the *A. mexicanum* PGC transcripts using the online miRNA database miRDB. We discovered 41 miRNAs present only in *A. mexicanum,* but none of them was shared in all PGC genes. Similarly, 50 miRNAs were specific only for *X. laevis* but were not shared in all its PGC genes (Supplementary Table S10).

### Conservation of vegetal and animal motifs

Analysis was carried out to determine if previously conserved animal and vegetal motifs detected in the oocyte of the *A. mexicanum* (Naraine et al., 2022) can also be seen preferentially enriched within the vegetal and animal transcripts during embryogenesis. Motifs were scanned using FIMO against the following datasets of developing *A. mexicanum* embryos: animal, vegetal, sectional profile altering, stage altering *de novo* and degraded DLTs.

Within the 3′ and 5’ UTR of transcripts from the *A. mexicanum* embryos, we observed the enrichment of the previously detected putative localization motifs that were found shared between both *A. mexicanum* and *X. laevis* oocytes and also those that were unique only for *A. mexicanum* oocytes (Supplementary Figure S3) (Naraine et al., 2022). The conserved motifs were detected only in the vegetally localized DLTs of *A. mexicanum* embryos and were mostly enriched with CAC or guanine-rich sequences.

The map of identified *de novo* motifs, miRNAs, RBPs and motifs from previous publication (Naraine et al., 2022) was assembled for selected PGC markers (Supplementary Figure S4).

## Discussion

Localization of transcripts established in the mature egg during oogenesis has a crucial role in asymmetric cell division during embryo development. Previously we identified thousands of DLTs in the maturating oocytes of *X. laevis* and *A. ruthenus* (Iegorova et al., 2022) and mature eggs of *X. laevis, A. mexicanum, A. ruthenus* and *D. rerio* (Naraine et al., 2022). The comparison of the evolutionary conservation of localized transcripts revealed many differences among analyzed species such as a low correlation in vegetal localization and dynamic changes in transcript levels. Based on these results we proposed that the development of different vertebrate species can be regulated in many ways.

To assess this, we performed a spatiotemporal analysis of transcript localization in early embryos of the urodele amphibian *A. mexicanum* and compared our results with available data from the anuran *X. laevis*. The transcriptome analysis in four developmental stages of *A. mexicanum* revealed very dynamic changes in RNA profiles and uncovered three divergent alterations—sectional profile alteration, *de novo* transcription and degradation. Early embryonic development is dependent on maternal RNA and protein storages in the absence of transcription, after which during MBT the transcription from the embryonic genome is initiated. In *X. laevis*, the MBT occurs after the 12th cycle of cell division, but in *A. mexicanum,* it can be either after the 9th cycle (Lefresne et al., 1998) or the 12th cycle of cell division (Jiang et al., 2017). Despite the first theories that transcription does not occur before MBT, later it has been shown in many species (e.g., *X. laevis* (Yang et al., 2002; Skirkanich et al., 2011), *M. musculus* (Bouniol et al., 1995; Aoki et al., 1997; Abe et al., 2018)) that a small number of genes can be transcribed even shortly after fertilization. In *A. mexicanum*, we identified more than 1.7% of maternal transcripts transcribed during the period from the fertilized egg until the early blastula (1K-cell) stage. Our findings support Jiang et al., 2017 and determine the onset of MBT after the 12th cycle of cell division. According to the GO analysis, *de novo* transcripts synthesized during this period have a structural and functional role in nascent cells. For example, *pi4ka* encodes kinase contributing to cell membrane synthesis (Wong and Cantley, 1994) and *epcam* is one of the main cell-to-cell adhesion molecules (Litvinov et al., 1994). Moreover, we identified *de novo* synthesis of *wee2*, whose zygotic expression was previously described in *X. laevis.* Leise and Mueller, 2002 proposed the possible mitosis-inhibiting role of Wee2 kinase in specific embryo tissues lacking proliferating cells.

The clearance of maternal transcripts before embryonic genome activation is required to regulate early embryo development and prepare the embryo for MBT. In *X. laevis*, deadenylation and transcript degradation can be mediated via RBPs recognizing specific RNA elements—deadenylation elements (Paillard et al., 1998) and AU-rich elements (Voeltz and Steitz, 1998)—or through zygotic miRNA (Lund et al., 2009; Koebernick et al., 2010). In *A. mexicanum,* we revealed the degradation of more than 1.5% of maternal transcripts. Previously, the degradation of several mRNAs (*wnt1, wnt5a, wnt5b*) during *A. mexicanum* early development was noticed (Caulet et al., 2010), but we are the first to describe the degradation in transcriptome-wide view in this model. Degraded maternal transcripts are enriched for GO terms related to cell cycle regulation, localization and the developmental process. For example, we have detected degradation of *actb,* which can be caused by actin disassembly at the cleaving egg (Field et al., 2019), and *ccna1*, which regulates cell cycle control (G1/S and G2/M). Degradation of the *ccna1* may be due to cyclin A1 redundancy given that the G1 phase is absent in the axolotl (Lefresne et al., 1998). Previously, Hamatani et al., 2004 described massive degradation of transcripts involved in the cell cycle also in *M. musculus* early embryos.

The PGC markers were previously identified to be localized to the vegetal pole in the eggs of *X. laevis* and *A. mexicanum* (Naraine et al., 2022), and have been corroborated by our research. While in *X. laevis*, the vegetal localization is established through the mitochondrial cloud (Kloc and Etkin, 1995), in *A. mexicanum*, this structure is probably absent and the mechanisms of mRNA localization remain unknown (Ikenishi and Nieuwkoop, 1978; Johnson et al., 2001). The interesting view on germ cell development in urodele was proposed by Škugor et al., 2016. In *A. mexicanum*, they described the functional loss of protein encoded by *velo1* which probably played an ancestral role in germ plasm assembly and proposed its role in germline formation in the ancestors of vertebrates. Therefore, they concluded that preformation is an ancestral mechanism and the inductive germ line determination occurred in vertebrates lacking germ plasm (e.g., urodeles, primates, rodents) due to convergent evolution. Our results may support this hypothesis because we detected the partial degradation of several PGC markers shortly after fertilization. Johnson et al. (2001) described the first appearance of *dazl,* another PGC marker, at stage 40 as well as the first formation of primordial germ cells. Therefore, to confirm, that the expression of PGC markers again starts after gastrulation (Johnson et al., 2001), we decided to detect 3 PGC markers (*grip2, dnd1* and *rbpms2*) and 1 vegetally localized unknown transcript using *in situ* hybridization. While *grip2* and *dnd1* were previously detected in PGCs (Tarbashevich et al., 2007; Koebernick et al., 2010)*, rbpms2* was detected only in oocytes and developing heart in *X.laevis* (Gerber et al., 1999; Zearfoss et al., 2004). Indeed, all of them was localized in the region of *A. mexicanum* presumptive germ cells at the stage 35 (Figure 5C), which is 5 stages earlier than reported by Johnson et al. (2001).

To take a deeper look into the processes of partial degradation of PGC markers, we analyzed their UTRs for a motif enrichment that may cause the degradation. We identified 1 motif (Motif 6, Figure 6A) within 3′UTR, which is exclusive for *A. mexicanum* PGC. It is interesting that the motif possibly binds PUM2 and ZFP36, which are RBPs involved in mRNA repression (Lai et al., 1999; Lai et al., 2003; Van Etten et al., 2012). Therefore, we concluded that this motif may potentially plays an important role in the partial degradation of the PGC marker RNAs after fertilization. Moreover, we identified 4 motifs exclusive in the 5′UTR of *A. mexicanum* PGCs mRNAs*,* but the role of these motifs is not known. Also, we identified several RBPs, potentially playing a role in degradation, which recognize binding sited within 3′UTR of PGCs mRNAs, but for these RBPs, the role in embryonic developmental degradation has not yet been described.

To find motifs specific for degradation or *de novo* synthesis, we searched motif enrichment within 3′ and 5′UTRs of maternal transcript in the degradation/*de novo* group, but no significant enrichment was observed. Moreover, we searched within animal or vegetal groups, to find enriched motifs for localization, but we did not find any significant enrichment. However, previously detected motifs enriched within the UTRs of vegetal and animally transcripts in the egg of the *A. mexicanum* (Naraine et al., 2022), revealed 5 motifs enriched within the 3′ and 5′UTRs of *A. mexicanum* embryos. These motifs are enriched with CAC core, a known localization motif, as well as some with guanine or cytosine-rich sequences, which may be potentially new localization elements.

The final mechanism on how the transcript can be regulated during early development is through profile alteration. We have determined sectional profile alteration in both vegetal and animal hemispheres. The formation or disruption of the vegetal profile was detected in a total of 2.3% DLTs. Out of these, 9% DLTs disrupt their vegetal profile due to transcript degradation and 3.4% DLTs create the profile due to *de novo* synthesis. The remaining 87.6% of transcripts show less than 3x fold change count across stages. Therefore, the sectional profile alteration for these remaining transcripts can be caused by either lower levels of degradation/*de novo* synthesis or active relocalization. GO term analysis performed on a subgroup containing transcripts whose vegetal localization gradually changed into uniform/slightly vegetal distribution, revealed their functions mainly in localization and protein folding. In *X. laevis*, for instance, Grip2 protein is indispensable for proper PGC migration (Kirilenko et al., 2008) and *kif4,* encoding microtubule motor protein*,* is essential for somatic cell division and its maternal paralog for meiotic division (Samwer et al., 2013; Ems-McClung et al., 2019). GO terms associated with transcripts whose vegetal profile rapidly changed into uniform/slightly vegetal distribution after the 1-cell stage, revealed their connection mainly with cellular components. As an example, *man2b1* (Malm and Nilssen, 2008) and *fuca1* (Willems et al., 1999) encode lysosomal enzymes and Sys1 protein is involved in protein trafficking (Behnia et al., 2004).

The alteration in animal profile was detected in almost 0.4% of DLTs. Of the total number of DLTs altering the animal profile, 78.7% of DLTs show less than 3x fold count change across individual stages. The animal alteration caused by transcript degradation was detected only in 1.9% DLTs, while *de novo* transcription was revealed in 19.4% DLTs. Transcripts forming animal profiles after the 1-cell stage are enriched for GO terms related to structural and functional components of the cell. Examples include *nek9,* encoding serine/threonine kinase, which plays an important role in mitotic spindle formation (Rapley et al., 2008) and Rrp12 protein important for ribosome assembly (Oeffinger et al., 2004).

Overall, our findings describing the regulation of maternal transcripts in early *A. mexicanum* embryos showed that after fertilization, maternal transcripts undergo multiple dynamic changes. These include alteration in localization or abundance and suggest that even related amphibians, such as *A. mexicanum* and *X. laevis*, can regulate their early development differently. The most prominent difference is probably the partial degradation of PGC markers, which indicates relevance for the differential development of germ cell establishment in the two amphibian orders. Our results support the necessity of cross-species comparison for a better understanding of aspects of the regulation of embryonic development.

## Data Availability

The datasets presented in this study can be found in online repositories. The names of the repository/repositories and accession number(s) can be found below: NCBI GEO under GSE240796.

## References

[B1] Abe K. Funaya S. Tsukioka D. Kawamura M. Suzuki Y. Suzuki M. (2018). Minor zygotic gene activation is essential for mouse preimplantation development. PNAS 115, E6780–E6788. 10.1073/pnas.1804309115 29967139 PMC6055165

[B2] Agius E. Oelgeschlager M. Wessely O. Kemp C. De Robertis E. M. (2000). Endodermal Nodal-related signals and mesoderm induction in Xenopus. Development 127, 1173–1183. 10.1242/dev.127.6.1173 10683171 PMC2292107

[B3] Aoki F. Worrad D. M. Schultz R. M. (1997). Regulation of transcriptional activity during the first and second cell cycles in the preimplantation mouse embryo. Dev. Biol. 181, 296–307. 10.1006/dbio.1996.8466 9013938

[B4] Bachvarova R. F. Masi T. Drum M. Parker N. Mason K. Patient R. (2004). Gene expression in the axolotl germ line: axdazl, axvh, axoct-4, and axkit. Dev. Dyn. 231, 871–880. 10.1002/dvdy.20195 15517581

[B5] Bailey T. L. (2021). STREME: accurate and versatile sequence motif discovery. Bioinformatics 37, 2834–2840. 10.1093/bioinformatics/btab203 33760053 PMC8479671

[B6] Behnia R. Panic B. Whyte J. R. C. Munro S. (2004). Targeting of the arf-like GTPase Arl3p to the Golgi requires N-terminal acetylation and the membrane protein Sys1p. Nat. Cell Biol. 6, 405–413. 10.1038/ncb1120 15077113

[B7] Benoit Bouvrette L. P. Bovaird S. Blanchette M. Lécuyer E. (2020). oRNAment: a database of putative RNA binding protein target sites in the transcriptomes of model species. Nucleic Acids Res. 48, D166-D173–D173. 10.1093/nar/gkz986 31724725 PMC7145663

[B8] Biswas A. Brown C. M. (2014). Scan for motifs: a webserver for the analysis of post-transcriptional regulatory elements in the 3′ untranslated regions (3′ UTRs) of mRNAs. BMC Bioinforma. 15, 174. 10.1186/1471-2105-15-174 PMC4067372 24909639

[B9] Bolger A. M. Lohse M. Usadel B. (2014). Trimmomatic: a flexible trimmer for Illumina sequence data. Bioinformatics 30, 2114–2120. 10.1093/bioinformatics/btu170 24695404 PMC4103590

[B10] Bordzilovskaya N. P. Dettlaff T. A. (1979). Table of stages of the normal development of axolotl embryos. 1-6. Axolotl Newsl. 7, 2–22.

[B11] Bouniol C. Nguyen E. Debey P. (1995). Endogenous transcription occurs at the 1-cell stage in the mouse embryo. Exp. Cell Res. 218, 57–62. 10.1006/excr.1995.1130 7537698

[B12] Brannon M. Gomperts M. Sumoy L. Moon R. T. Kimelman D. (1997). A beta-catenin/XTcf-3 complex binds to the siamois promoter to regulate dorsal axis specification in Xenopus. Genes Dev. 11, 2359–2370. 10.1101/gad.11.18.2359 9308964 PMC316518

[B13] Bray N. L. Pimentel H. Melsted P. Pachter L. (2016). Near-optimal probabilistic RNA-seq quantification. Nat. Biotechnol. 34, 525–527. 10.1038/nbt.3519 27043002

[B114] Caulet S. Pelczar H. Andéol Y. (2010). Multiple sequences and factors are involved in stability/degradation of Awnt-1, Awnt-5A and Awnt-5B mRNAs during axolotl development. Development growth and differentiation 52 (2) 209–222. 10.1111/j.1440-169X.2009.01156.x 20151991

[B14] Chan A. P. Kloc M. Etkin L. D. (1999). Fatvg encodes a new localized RNA that uses a 25-nucleotide element (FVLE1) to localize to the vegetal cortex of Xenopus oocytes. Development 126, 4943–4953. 10.1242/dev.126.22.4943 10529413

[B15] Chang P. Torres J. Lewis R. A. Mowry K. L. Houliston E. King M. L. (2004). Localization of RNAs to the mitochondrial cloud in Xenopus oocytes through entrapment and association with endoplasmic reticulum. Mol. Biol. Cell. 15, 4669–4681. 10.1091/mbc.e04-03-0265 15292452 PMC519158

[B16] Chen Y. Wang X. (2020). miRDB: an online database for prediction of functional microRNA targets. Nucleic Acids Res. 48, D127-D131–D131. 10.1093/nar/gkz757 31504780 PMC6943051

[B17] Clauβen M. Tarbashevich K. Pieler T. (2011). Functional dissection of the RNA signal sequence responsible for vegetal localization of XGrip2.1 mRNA in Xenopus oocytes. RNA Biol. 8, 873–882. 10.4161/rna.8.5.16028 21788733

[B18] Cook K. B. Kazan H. Zuberi K. Morris Q. Hughes T. R. (2011). RBPDB: a database of RNA-binding specificities. Nucleic Acids Res. 39, D301–D308. 10.1093/nar/gkq1069 21036867 PMC3013675

[B19] Dassi E. Re A. Leo S. Tebaldi T. Pasini L. Peroni D. (2014). AURA 2: empowering discovery of post-transcriptional networks. Transl. (Austin) 2, e27738. 10.4161/trla.27738 PMC4705823 26779400

[B20] De Domenico E. Owens N. D. L. Grant I. M. Gomes-Faria R. Gilchrist M. J. (2015). Molecular asymmetry in the 8-cell stage Xenopus tropicalis embryo described by single blastomere transcript sequencing. Dev. Biol. 408, 252–268. 10.1016/j.ydbio.2015.06.010 26100918 PMC4684228

[B21] Delarue M. Sáez F. J. Johnson K. E. Boucaut J. C. (1997). Fates of the blastomeres of the 32-cell stage *Pleurodeles waltl* embryo. Dev. Dyn. 210, 236–248. 10.1002/(SICI)1097-0177(199711)210:3<236::AID-AJA5>3.0.CO;2-H 9389450

[B22] Deshler J. O. Highett M. I. Schnapp B. J. (1997). Localization of Xenopus Vg1 mRNA by vera protein and the endoplasmic reticulum. Science 276, 1128–1131. 10.1126/science.276.5315.1128 9148809

[B23] Dumont J. (1972). Oogenesis in *Xenopus laevis* (Daudin). I. Stages of oocyte development in laboratory maintained animals. J. Morphol. 136, 153–179. 10.1002/jmor.1051360203 4109871

[B24] Ems-McClung S. C. Emch M. Zhang S. Mahnoor S. Weaver L. N. Walczak C. E. (2019). RanGTP induces an effector gradient of XCTK2 and importin α/β for spindle microtubule cross-linking. J. Cell Biol. 219, e201906045. 10.1083/jcb.201906045 PMC7041689 31865374

[B25] Field C. M. Pelletier J. F. Mitchison T. J. (2019). Disassembly of actin and keratin networks by aurora B kinase at the midplane of cleaving *Xenopus laevis* eggs. Curr. Biol. 29, 1999–2008. 10.1016/j.cub.2019.05.016 31178324 PMC6639026

[B26] Flachsova M. Sindelka R. Kubista M. (2013). Single blastomere expression profiling of *Xenopus laevis* embryos of 8 to 32-cells reveals developmental asymmetry. Sci. Rep. 3, 2278. 10.1038/srep02278 23880666 PMC3721081

[B27] Forristall C. Pondel M. Chen L. King M. L. (1995). Patterns of localization and cytoskeletal association of two vegetally localized RNAs, Vg1 and Xcat-2. Dev 121, 201–208. 10.1242/dev.121.1.201 7867501

[B28] Fuentes R. Mullins M. C. Fernández J. (2018). Formation and dynamics of cytoplasmic domains and their genetic regulation during the zebrafish oocyte-to-embryo transition. Mech. Dev. 154, 259–269. 10.1016/j.mod.2018.08.001 30077623

[B29] Ge X. Grotjahn D. Welch E. Lyman-Gingerich J. Holguin C. Dimitrova E. (2014). Hecate/Grip2a acts to reorganize the cytoskeleton in the symmetry-breaking event of embryonic Axis induction. PLOS Genet. 10, e1004422. 10.1371/journal.pgen.1004422 24967891 PMC4072529

[B30] Gerber W. V. Yatskievych T. A. Antin P. B. Correia K. M. Conlon R. A. Krieg P. A. (1999). The RNA-binding protein gene, hermes, is expressed at high levels in the developing heart. Mech. Dev. 80, 77–86. 10.1016/s0925-4773(98)00195-6 10096065

[B31] Grant C. E. Bailey T. L. Noble W. S. (2011). FIMO: scanning for occurrences of a given motif. Bioinformatics 27, 1017–1018. 10.1093/bioinformatics/btr064 21330290 PMC3065696

[B32] Guarracino A. Pepe G. Ballesio F. Adinolfi M. Pietrosanto M. Sangiovanni E. (2021). BRIO: a web server for RNA sequence and structure motif scan. Nucleic Acids Res. 49, W67–W71. 10.1093/nar/gkab400 34038531 PMC8262756

[B33] Gupta S. Stamatoyannopoulos J. A. Bailey T. L. Noble W. S. (2007). Quantifying similarity between motifs. Genome Biol. 8, R24. 10.1186/gb-2007-8-2-r24 17324271 PMC1852410

[B34] Hamatani T. Carter M. G. Sharov A. A. Ko M. S. H. (2004). Dynamics of global gene expression changes during mouse preimplantation development. Dev. Cell 6, 117–131. 10.1016/s1534-5807(03)00373-3 14723852

[B35] Hashimoto Y. Maegawa S. Nagai T. Yamaha E. Suzuki H. Yasuda K. (2004). Localized maternal factors are required for zebrafish germ cell formation. Dev. Biol. 268, 152–161. 10.1016/j.ydbio.2003.12.013 15031112

[B36] Heasman J. Quarmby J. Wylie C. C. (1984). The mitochondrial cloud of Xenopus oocytes: the source of germinal granule material. Dev. Biol. 105, 458–469. 10.1016/0012-1606(84)90303-8 6541166

[B37] Horvay K. Claußen M. Katzer M. Landgrebe J. Pieler T. (2006). Xenopus Dead end mRNA is a localized maternal determinant that serves a conserved function in germ cell development. Dev. Biol. 291, 1–11. 10.1016/j.ydbio.2005.06.013 16448642

[B38] Houston D. W. Zhang J. Maines J. Z. Wasserman S. A. King M. L. (1998). A Xenopus DAZ-like gene encodes an RNA component of germ plasm and is a functional homologue of Drosophila boule. Development 125, 171–180. 10.1242/dev.125.2.171 9486791

[B39] Howley C. Ho R. K. (2000). mRNA localization patterns in zebrafish oocytes. Mech. Dev. 92, 305–309. 10.1016/s0925-4773(00)00247-1 10727871

[B40] Iegorova V. Naraine R. Psenicka M. Zelazowska M. Sindelka R. (2022). Comparison of RNA localization during oogenesis within *Acipenser ruthenus* and *Xenopus laevis* . Front. Cell. Dev. Biol. 10, 982732. 10.3389/fcell.2022.982732 36204678 PMC9531136

[B41] Ikenishi K. Nieuwkoop P. D. (1978). Location and ultrastructure of primordial germ cells (PGCs) in *Ambystoma mexicanum* . Dev. Growth Differ. 20, 1–9. 10.1111/j.1440-169X.1978.00001.x 37281741

[B42] Jiang P. Nelson J. D. Leng N. Collins M. Swanson S. Dewey C. N. (2017). Analysis of embryonic development in the unsequenced axolotl: waves of transcriptomic upheaval and stability. Dev. Biol. Xenopus Genomes 426, 143–154. 10.1016/j.ydbio.2016.05.024 PMC5272911 27475628

[B43] Johnson A. D. Bachvarova R. F. Drum M. Masi T. (2001). Expression of axolotl DAZL RNA, a marker of germ plasm: widespread maternal RNA and onset of expression in germ cells approaching the gonad. Dev. Biol. 234, 402–415. 10.1006/dbio.2001.0264 11397009

[B44] Kirilenko P. Weierud F. K. Zorn A. M. Woodland H. R. (2008). The efficiency of Xenopus primordial germ cell migration depends on the germplasm mRNA encoding the PDZ domain protein Grip2. Differentiation 76, 392–403. 10.1111/j.1432-0436.2007.00229.x 17924960

[B45] Kloc M. Etkin L. D. (1995). Two distinct pathways for the localization of RNAs at the vegetal cortex in Xenopus oocytes. Development 121, 287–297. 10.1242/dev.121.2.287 7539356

[B46] Knaut H. Pelegri F. Bohmann K. Schwarz H. Nüsslein-Volhard C. (2000). Zebrafish vasa RNA but not its protein is a component of the germ plasm and segregates asymmetrically before germline specification. J. Cell Biol. 149, 875–888. 10.1083/jcb.149.4.875 10811828 PMC2174565

[B47] Koebernick K. Loeber J. Arthur P. K. Tarbashevich K. Pieler T. (2010). Elr-type proteins protect Xenopus Dead end mRNA from miR-18-mediated clearance in the soma. PNAS 107, 16148–16153. 10.1073/pnas.1004401107 20805475 PMC2941339

[B48] Kofron M. Demel T. Xanthos J. Lohr J. Sun B. Sive H. (1999). Mesoderm induction in Xenopus is a zygotic event regulated by maternal VegT via TGFbeta growth factors. Development 126, 5759–5770. 10.1242/dev.126.24.5759 10572051

[B49] Kopylova E. Noé L. Touzet H. (2012). SortMeRNA: fast and accurate filtering of ribosomal RNAs in metatranscriptomic data. Bioinformatics 28, 3211–3217. 10.1093/bioinformatics/bts611 23071270

[B50] Kosaka K. Kawakami K. Sakamoto H. Inoue K. (2007). Spatiotemporal localization of germ plasm RNAs during zebrafish oogenesis. Mech. Dev. 124, 279–289. 10.1016/j.mod.2007.01.003 17293094

[B51] Lai W. S. Carballo E. Strum J. R. Kennington E. A. Phillips R. S. Blackshear P. J. (1999). Evidence that tristetraprolin binds to AU-rich elements and promotes the deadenylation and destabilization of tumor necrosis factor alpha mRNA. Mol. Cell Biol. 19, 4311–4323. 10.1128/mcb.19.6.4311 10330172 PMC104391

[B52] Lai W. S. Kennington E. A. Blackshear P. J. (2003). Tristetraprolin and its family members can promote the cell-free deadenylation of AU-rich element-containing mRNAs by poly(A) ribonuclease. Mol. Cell Biol. 23, 3798–3812. 10.1128/mcb.23.11.3798-3812.2003 12748283 PMC155217

[B53] Laurent M. N. Blitz I. L. Hashimoto C. Rothbächer U. Cho K. W. (1997). The Xenopus homeobox gene twin mediates Wnt induction of goosecoid in establishment of Spemann’s organizer. Development 124, 4905–4916. 10.1242/dev.124.23.4905 9428427

[B54] Lechner M. Findeiss S. Steiner L. Marz M. Stadler P. F. Prohaska S. J. (2011). Proteinortho: detection of (co-)orthologs in large-scale analysis. BMC Bioinforma. 12, 124. 10.1186/1471-2105-12-124 PMC3114741 21526987

[B55] Lefresne J. Andéol Y. Signoret J. (1998). Evidence for introduction of a variable G1 phase at the midblastula transition during early development in axolotl. Dev. Growth Differ. 40, 497–508. 10.1046/j.1440-169x.1998.t01-3-00004.x 9783475

[B56] Leise W. F. Mueller P. R. (2002). Multiple Cdk1 inhibitory kinases regulate the cell cycle during development. Dev. Biol. 249, 156–173. 10.1006/dbio.2002.0743 12217326

[B115] Levin M. Thorlin T. Robinson K. R. Nogi T. Mercola M. (2002). Asymmetries in H+/K+-ATPase and cell membrane potentials comprise a very early step in left-right patterning. Development 111 (1) 77–89. 10.1016/S0092-8674(02)00939-X 12372302

[B57] Litvinov S. V. Velders M. P. Bakker H. A. Fleuren G. J. Warnar S. O. (1994). Ep-CAM: a human epithelial antigen is a homophilic cell-cell adhesion molecule. J. Cell Biol. 125, 437–446. 10.1083/jcb.125.2.437 8163559 PMC2120036

[B58] Love M. I. Huber W. Anders S. (2014). Moderated estimation of fold change and dispersion for RNA-seq data with DESeq2. Genome Biol. 15, 550. 10.1186/s13059-014-0550-8 25516281 PMC4302049

[B59] Lu F.-I. Thisse C. Thisse B. (2011). Identification and mechanism of regulation of the zebrafish dorsal determinant. Proc. Natl. Acad. Sci. 108, 15876–15880. 10.1073/pnas.1106801108 21911385 PMC3179059

[B60] Lund E. Liu M. Hartley R. S. Sheets M. D. Dahlberg J. E. (2009). Deadenylation of maternal mRNAs mediated by miR-427 in *Xenopus laevis* embryos. RNA 15, 2351–2363. 10.1261/rna.1882009 19854872 PMC2779678

[B61] Lundmark C. (1986). Role of bilateral zones of ingressing superficial cells during gastrulation of *Ambystoma mexicanum* . Development 97, 47–62. 10.1242/dev.97.1.47 3794603

[B62] Lustig K. D. Kroll K. L. Sun E. E. Kirschner M. W. (1996). Expression cloning of a Xenopus T-related gene (Xombi) involved in mesodermal patterning and blastopore lip formation. Development 122, 4001–4012. 10.1242/dev.122.12.4001 9012520

[B63] McLeay R. C. Bailey T. L. (2010). Motif enrichment analysis: a unified framework and an evaluation on ChIP data. BMC Bioinforma. 11, 165. 10.1186/1471-2105-11-165 PMC2868005 20356413

[B64] MacArthur H. Houston D. W. Bubunenko M. Mosquera L. King M. L. (2000). DEADSouth is a germ plasm specific DEAD-box RNA helicase in Xenopus related to eIF4A. Mech. Dev. 95, 291–295. 10.1016/s0925-4773(00)00357-9 10906480

[B65] Mahowald A. P. Hennen S. (1971). Ultrastructure of the “germ plasm” in eggs and embryos of *Rana pipiens* . Dev. Biol. 24, 37–53. 10.1016/0012-1606(71)90045-5 4107682

[B66] Malm D. Nilssen Ø. (2008). Alpha-mannosidosis. Orphanet J. Rare Dis. 3, 21. 10.1186/1750-1172-3-21 18651971 PMC2515294

[B67] Mattei E. Pietrosanto M. Ferrè F. Helmer-Citterich M. (2015). Web-beagle: a web server for the alignment of RNA secondary structures. Nucleic Acids Res. 43, W493–W497. 10.1093/nar/gkv489 25977293 PMC4489221

[B68] Melton D. A. (1987). Translocation of a localized maternal mRNA to the vegetal pole of Xenopus oocytes. Nature 328, 80–82. 10.1038/328080a0 3600777

[B69] Moody S. A. (1987a). Fates of the blastomeres of the 16-cell stage Xenopus embryo. Dev. Biol. 119, 560–578. 10.1016/0012-1606(87)90059-5 3803718

[B70] Moody S. A. (1987b). Fates of the blastomeres of the 32-cell-stage Xenopus embryo. Dev. Biol. 122, 300–319. 10.1016/0012-1606(87)90296-x 3596014

[B71] Naraine R. Iegorova V. Abaffy P. Franek R. Soukup V. Psenicka M. (2022). Evolutionary conservation of maternal RNA localization in fishes and amphibians revealed by TOMO-Seq. Dev. Biol. 489, 146–160. 10.1016/j.ydbio.2022.06.013 35752299

[B72] Nath K. Boorech J. L. Beckham Y. M. Burns M. M. Elinson R. P. (2005). Status of RNAs, localized in *Xenopus laevis* oocytes, in the frogs *Rana pipiens* and Eleutherodactylus coqui. J. Exp. Zool. 304B, 28–39. 10.1002/jez.b.21020 15515051

[B73] Nishita M. Hashimoto M. K. Ogata S. Laurent M. N. Ueno N. Shibuya H. (2000). Interaction between Wnt and TGF-beta signalling pathways during formation of Spemann’s organizer. Nature 403, 781–785. 10.1038/35001602 10693808

[B116] Nowoshilow S. Tanaka E. M. (2020). Introducing www.axolotl-omics.org – an integrated -omics data portal for the axolotl research community. Exp. Cell Res 394 (1) 112143. 10.1016/j.yexcr.2020.112143 32540400

[B74] O’Brien J. Hayder H. Zayed Y. Peng C. (2018). Overview of MicroRNA biogenesis, mechanisms of actions, and circulation. Front. Endocrinol. 9, 402. 10.3389/fendo.2018.00402 PMC6085463 30123182

[B75] Oeffinger M. Dlakić M. Tollervey D. (2004). A pre-ribosome-associated HEAT-repeat protein is required for export of both ribosomal subunits. Genes Dev. 18, 196–209. 10.1101/gad.285604 14729571 PMC324425

[B76] Paillard L. Omilli F. Legagneux V. Bassez T. Maniey D. Osborne H. B. (1998). EDEN and EDEN-BP, a cis element and an associated factor that mediate sequence-specific mRNA deadenylation in Xenopus embryos. EMBO J. 17, 278–287. 10.1093/emboj/17.1.278 9427761 PMC1170378

[B77] Pantano L. Hutchinson J. Barrera V. Piper M. Khetani R. Daily K. (2023). DEGreport: Report of DEG analysis. Available at: http://lpantano.github.io/DEGreport/ .

[B78] Pietrosanto M. Adinolfi M. Casula R. Ausiello G. Ferrè F. Helmer-Citterich M. (2018). BEAM web server: a tool for structural RNA motif discovery. Bioinformatics 34, 1058–1060. 10.1093/bioinformatics/btx704 29095974 PMC5860439

[B79] Rapley J. Nicolàs M. Groen A. Regué L. Bertran M. T. Caelles C. (2008). The NIMA-family kinase Nek6 phosphorylates the kinesin Eg5 at a novel site necessary for mitotic spindle formation. J. Cell Sci. 121, 3912–3921. 10.1242/jcs.035360 19001501 PMC4066659

[B80] Raudvere U. Kolberg L. Kuzmin I. Arak T. Adler P. Peterson H. (2019). g:Profiler: a web server for functional enrichment analysis and conversions of gene lists (2019 update). Nucleic Acids Res. 47, W191-W198–W198. 10.1093/nar/gkz369 31066453 PMC6602461

[B81] Ray D. Kazan H. Cook K. B. Weirauch M. T. Najafabadi H. S. Li X. (2013). A compendium of RNA-binding motifs for decoding gene regulation. Nature 499, 172–177. 10.1038/nature12311 23846655 PMC3929597

[B82] Samwer M. Dehne H.-J. Spira F. Kollmar M. Gerlich D. W. Urlaub H. (2013). The nuclear F-actin interactome of Xenopus oocytes reveals an actin-bundling kinesin that is essential for meiotic cytokinesis. EMBO J. 32, 1886–1902. 10.1038/emboj.2013.108 23727888 PMC3981176

[B83] Schreckenberg G. M. Jacobson A. G. (1975). Normal stages of development of the axolotl. *Ambystoma mexicanum* . Dev. Biol. 42, 391–400. 10.1016/0012-1606(75)90343-7 1167837

[B84] Sekula M. Datta S. Datta S. (2017). optCluster: an R Package for determining the optimal clustering algorithm. Bioinformation 13, 101–103. 10.6026/97320630013101 28584451 PMC5450252

[B85] Sindelka R. Abaffy P. Qu Y. Tomankova S. Sidova M. Naraine R. (2018). Asymmetric distribution of biomolecules of maternal origin in the *Xenopus laevis* egg and their impact on the developmental plan. Sci. Rep. 8, 8315. 10.1038/s41598-018-26592-1 29844480 PMC5974320

[B86] Sindelka R. Jonák J. Hands R. Bustin S. A. Kubista M. (2008). Intracellular expression profiles measured by real-time PCR tomography in the *Xenopus laevis* oocyte. Nucleic Acids Res. 36, 387–392. 10.1093/nar/gkm1024 18039714 PMC2241880

[B87] Sindelka R. Sidova M. Svec D. Kubista M. (2010). Spatial expression profiles in the *Xenopus laevis* oocytes measured with qPCR tomography. Methods, Xenopus Oocytes as Exp. Syst. 51, 87–91. 10.1016/j.ymeth.2009.12.011 20051264

[B88] Skirkanich J. Luxardi G. Yang J. Kodjabachian L. Klein P. S. (2011). An essential role for transcription before the MBT in *Xenopus laevis* . Dev. Biol. 357, 478–491. 10.1016/j.ydbio.2011.06.010 21741375 PMC3164747

[B89] Škugor A. Tveiten H. Johnsen H. Andersen Ø. (2016). Multiplicity of Buc copies in Atlantic salmon contrasts with loss of the germ cell determinant in primates, rodents and axolotl. BMC Evol. Biol. 16, 232. 10.1186/s12862-016-0809-7 27784263 PMC5080839

[B90] Soukup V. Tazaki A. Yamazaki Y. Pospisilova A. Epperlein H.-H. Tanaka E. M. (2021). Oral and palatal dentition of axolotl arises from a common tooth-competent zone along the ecto-endodermal boundary. Front. Cell. Dev. Biol. 8, 622308. 10.3389/fcell.2020.622308 33505974 PMC7829593

[B91] Stennard F. Carnac G. Gurdon J. B. (1996). The Xenopus T-box gene, Antipodean, encodes a vegetally localised maternal mRNA and can trigger mesoderm formation. Development 122, 4179–4188. 10.1242/dev.122.12.4179 9012537

[B92] Supek F. Bošnjak M. Škunca N. Šmuc T. (2011). REVIGO summarizes and visualizes long lists of gene ontology terms. PLOS ONE 6, e21800. 10.1371/journal.pone.0021800 21789182 PMC3138752

[B93] Tam P. P. Zhou S. X. (1996). The allocation of epiblast cells to ectodermal and germ-line lineages is influenced by the position of the cells in the gastrulating mouse embryo. Dev. Biol. 178, 124–132. 10.1006/dbio.1996.0203 8812114

[B94] Tarbashevich K. Koebernick K. Pieler T. (2007). XGRIP2.1 is encoded by a vegetally localizing, maternal mRNA and functions in germ cell development and anteroposterior PGC positioning in *Xenopus laevis* . Dev. Biol. 311, 554–565. 10.1016/j.ydbio.2007.09.012 17936745

[B95] Theusch E. V. Brown K. J. Pelegri F. (2006). Separate pathways of RNA recruitment lead to the compartmentalization of the zebrafish germ plasm. Dev. Biol. 292, 129–141. 10.1016/j.ydbio.2005.12.045 16457796

[B96] Tran L. D. Hino H. Quach H. Lim S. Shindo A. Mimori-Kiyosue Y. (2012). Dynamic microtubules at the vegetal cortex predict the embryonic axis in zebrafish. Development 139, 3644–3652. 10.1242/dev.082362 22949618

[B97] Untergasser A. Cutcutache I. Koressaar T. Ye J. Faircloth B. C. Remm M. (2012). Primer3--new capabilities and interfaces. Nucleic Acids Res. 40, e115. 10.1093/nar/gks596 22730293 PMC3424584

[B98] Van Etten J. Schagat T. L. Hrit J. Weidmann C. A. Brumbaugh J. Coon J. J. (2012). Human Pumilio proteins recruit multiple deadenylases to efficiently repress messenger RNAs. J. Biol. Chem. 287, 36370–36383. 10.1074/jbc.M112.373522 22955276 PMC3476303

[B99] Vaur S. Montreau N. Dautry F. Andeol Y. (2003). Differential post-transcriptional regulations of wnt mRNAs upon axolotl meiotic maturation. Int. J. Dev. Biol. 46, 731–739. 12216985

[B100] Venkatarama T. Lai F. Luo X. Zhou Y. Newman K. King M. L. (2010). Repression of zygotic gene expression in the Xenopus germline. Development 137, 651–660. 10.1242/dev.038554 20110330 PMC2827618

[B101] Vincent J.-P. Oster G. F. Gerhart J. C. (1986). Kinematics of gray crescent formation in Xenopus eggs: the displacement of subcortical cytoplasm relative to the egg surface. Dev. Biol. 113, 484–500. 10.1016/0012-1606(86)90184-3 3949075

[B102] Vincent J.-P. Scharf S. R. Gerhart J. C. (1987). Subcortical rotation in Xenopus eggs: A preliminary study of its mechanochemical basis. Cell Motil. 8, 143–154. 10.1002/cm.970080206 3690686

[B103] Vinot S. Le T. Ohno S. Pawson T. Maro B. Louvet-Vallée S. (2005). Asymmetric distribution of PAR proteins in the mouse embryo begins at the 8-cell stage during compaction. Dev. Biol. 282, 307–319. 10.1016/j.ydbio.2005.03.001 15950600

[B104] Voeltz G. K. Steitz J. A. (1998). AUUUA sequences direct mRNA deadenylation uncoupled from decay during Xenopus early development. Mol. Cell. Biol. 18, 7537–7545. 10.1128/mcb.18.12.7537 9819439 PMC109334

[B105] Weidinger G. Stebler J. Slanchev K. Dumstrei K. Wise C. Lovell-Badge R. (2003). dead end, a novel vertebrate germ plasm component, is required for zebrafish primordial germ cell migration and survival. Curr. Biol. 13, 1429–1434. 10.1016/s0960-9822(03)00537-2 12932328

[B106] Welch E. Pelegri F. (2015). Cortical depth and differential transport of vegetally localized dorsal and germ line determinants in the zebrafish embryo. BioArchitecture 5, 13–26. 10.1080/19490992.2015.1080891 PMC4832442 26528729

[B107] Whitington P. M. D. Dixon K. E. (1975). Quantitative studies of germ plasm and germ cells during early embryogenesis of *Xenopus laevis* . Development 33, 57–74. 10.1242/dev.33.1.57 1151270

[B108] Willems P. J. Seo H.-C. Coucke P. Tonlorenzi R. O’Brien J. S. (1999). Spectrum of mutations in fucosidosis. Eur. J. Hum. Genet. 7, 60–67. 10.1038/sj.ejhg.5200272 10094192

[B109] Wong K. Cantley L. C. (1994). Cloning and characterization of a human phosphatidylinositol 4-kinase. J. Biol. Chem. 269, 28878–28884. 10.1016/s0021-9258(19)61989-7 7961848

[B110] Yang J. Tan C. Darken R. S. Wilson P. A. Klein P. S. (2002). Beta-catenin/Tcf-regulated transcription prior to the midblastula transition. Development 129, 5743–5752. 10.1242/dev.00150 12421713

[B111] Zearfoss N. R. Chan A. P. Wu C. F. Kloc M. Etkin L. D. (2004). Hermes is a localized factor regulating cleavage of vegetal blastomeres in *Xenopus laevis* . Dev. Biol. 267, 60–71. 10.1016/j.ydbio.2003.10.032 14975717

[B112] Zhang J. King M. L. (1996). Xenopus VegT RNA is localized to the vegetal cortex during oogenesis and encodes a novel T-box transcription factor involved in mesodermal patterning. Development 122, 4119–4129. 10.1242/dev.122.12.4119 9012531

[B113] Zhou Y. King M. L. (1996). Localization of Xcat-2 RNA, a putative germ plasm component, to the mitochondrial cloud in Xenopus stage I oocytes. Development 122, 2947–2953. 10.1242/dev.122.9.2947 8787767

