# Supplementary material for: RNA localization during early development of the axolotl
Source: Front Cell Dev Biol. 2023 Oct 19;11:1260795. doi: 10.3389/fcell.2023.1260795 (PMC10620976; doi:10.3389/fcell.2023.1260795)
Supplement: Supplementary file 1 [file Presentation1.PPTX]

## Slide 1
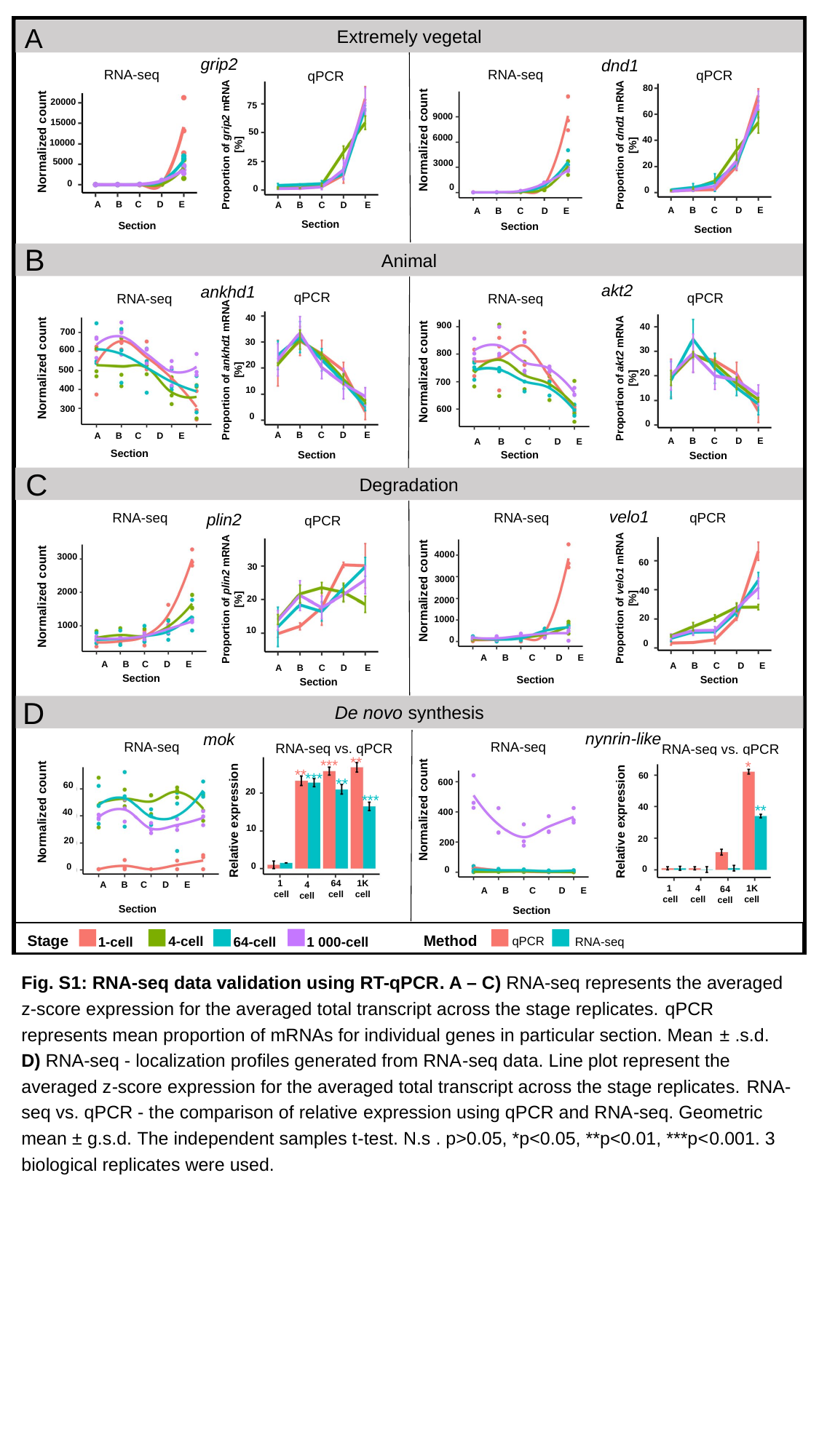

A
Extremely vegetal
grip2
dnd1
RNA-seq
RNA-seq
qPCR
qPCR
80
20000
75
60
9000
15000
50
6000
40
Normalized count
Normalized count
10000
Proportion of grip2 mRNA [%]
Proportion of dnd1 mRNA [%]
5000
25
3000
20
0
0
0
0
 A B C D E
 A B C D E
 A B C D E
 A B C D E
Section
Section
Section
Section
B
Animal
akt2
ankhd1
qPCR
qPCR
RNA-seq
RNA-seq
40
900
40
700
30
600
30
800
20
Normalized count
Proportion of ankhd1 mRNA [%]
500
Normalized count
20
Proportion of akt2 mRNA [%]
700
400
10
10
600
300
0
0
 A B C D E
 A B C D E
 A B C D E
 A B C D E
Section
Section
Section
Section
C
Degradation
velo1
plin2
RNA-seq
RNA-seq
qPCR
qPCR
4000
3000
60
30
3000
Normalized count
40
2000
Normalized count
Proportion of velo1 mRNA [%]
20
Proportion of plin2 mRNA [%]
2000
20
1000
1000
10
0
0
 A B C D E
 A B C D E
 A B C D E
 A B C D E
Section
Section
Section
Section
D
De novo synthesis
nynrin-like
mok
RNA-seq
RNA-seq
RNA-seq vs. qPCR
RNA-seq vs. qPCR
**
***
*
**
***
60
**
600
60
20
***
**
40
Normalized count
Normalized count
40
400
Relative expression
Relative expression
10
20
20
200
0
0
0
0
1
cell
64
cell
1K
cell
4
cell
 A B C D E
1
cell
4
cell
1K
cell
64
cell
 A B C D E
Section
Section
Section
Section
Section
 Method
 Stage
 4-cell
 64-cell
 1-cell
 1 000-cell
qPCR
RNA-seq
Fig. S1: RNA-seq data validation using RT-qPCR. A – C) RNA-seq represents the averaged z-score expression for the averaged total transcript across the stage replicates. qPCR represents mean proportion of mRNAs for individual genes in particular section. Mean ± .s.d. D) RNA-seq - localization profiles generated from RNA-seq data. Line plot represent the averaged z-score expression for the averaged total transcript across the stage replicates. RNA-seq vs. qPCR - the comparison of relative expression using qPCR and RNA-seq. Geometric mean ± g.s.d. The independent samples t-test. N.s . p>0.05, *p<0.05, **p<0.01, ***p<0.001. 3 biological replicates were used.

## Slide 2
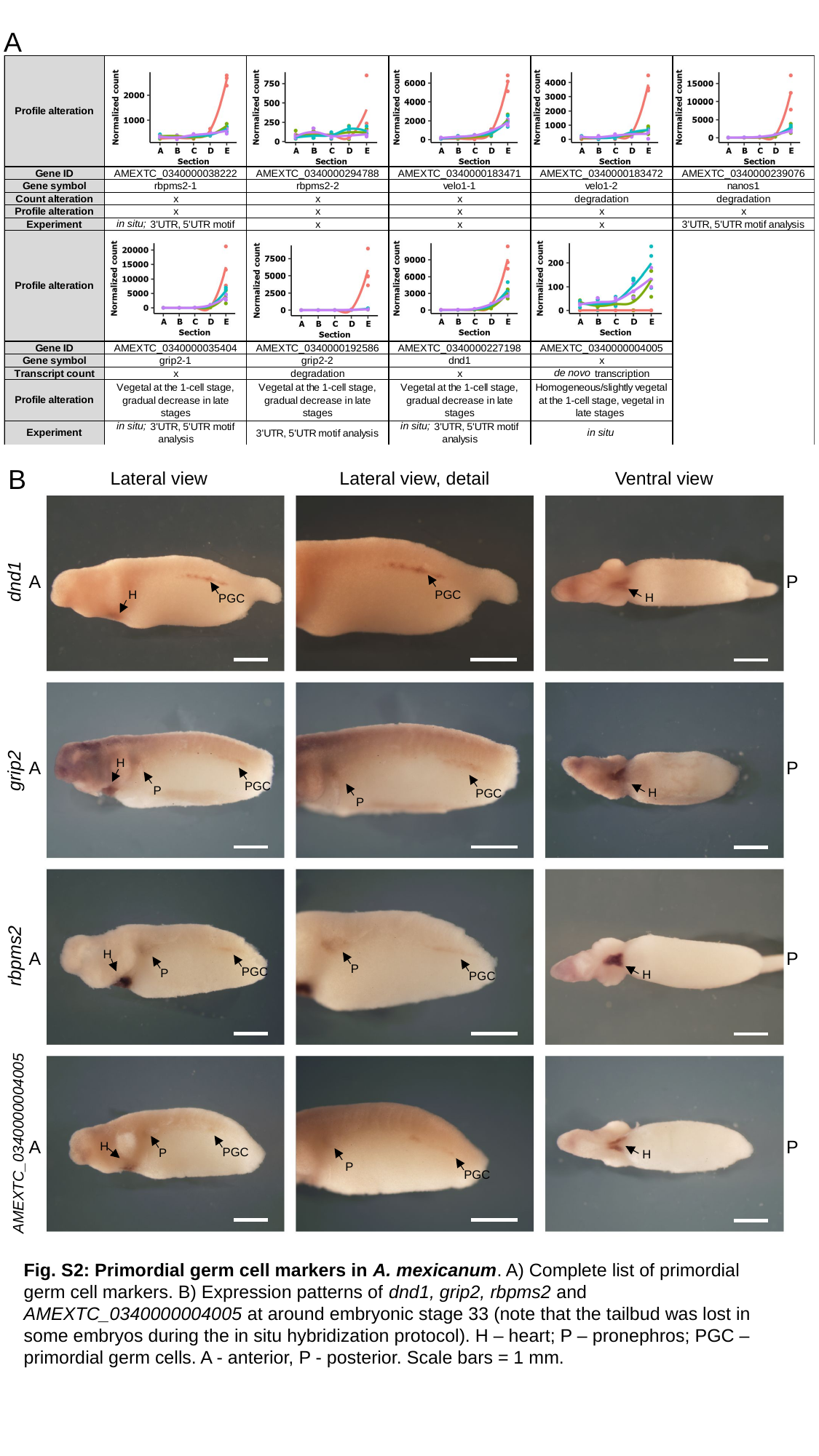

A
B
Lateral view, detail
Ventral view
Lateral view
dnd1
PGC
H
PGC
H
H
grip2
PGC
P
PGC
P
rbpms2
H
P
PGC
P
PGC
AMEXTC_0340000004005
H
PGC
P
P
PGC
A
P
P
A
H
A
P
H
A
P
H
Fig. S2: Primordial germ cell markers in A. mexicanum. A) Complete list of primordial germ cell markers. B) Expression patterns of dnd1, grip2, rbpms2 and AMEXTC_0340000004005 at around embryonic stage 33 (note that the tailbud was lost in some embryos during the in situ hybridization protocol). H – heart; P – pronephros; PGC – primordial germ cells. A - anterior, P - posterior. Scale bars = 1 mm.

## Slide 3
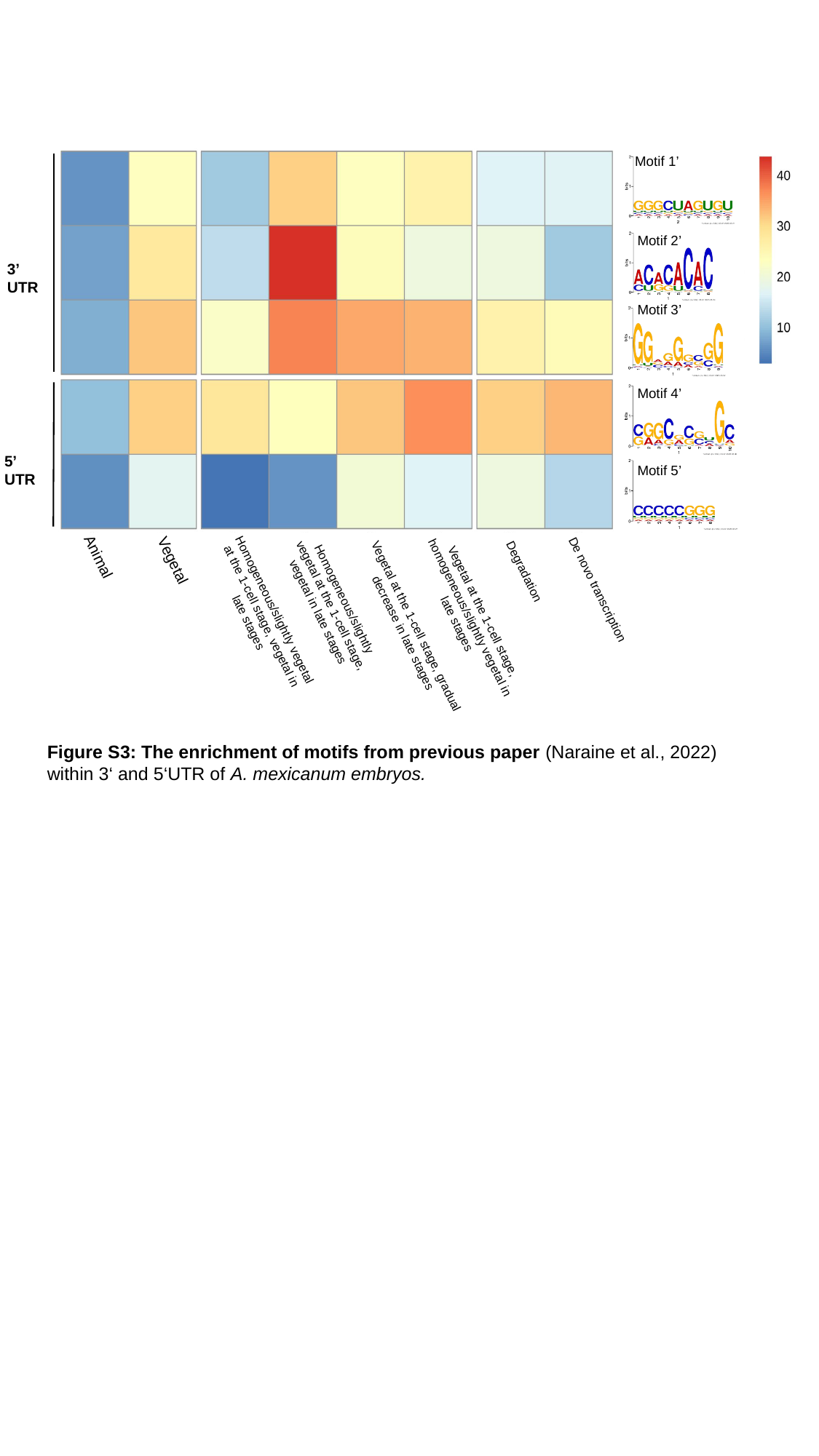

Motif 1’
Motif 2’
3’ UTR
Motif 3’
Motif 4’
5’ UTR
Motif 5’
Animal
Vegetal
Degradation
De novo transcription
Homogeneous/slightly vegetal at the 1-cell stage, vegetal in late stages
Homogeneous/slightly vegetal at the 1-cell stage, vegetal in late stages
Vegetal at the 1-cell stage, homogeneous/slightly vegetal in late stages
Vegetal at the 1-cell stage, gradual decrease in late stages
Figure S3: The enrichment of motifs from previous paper (Naraine et al., 2022) within 3‘ and 5‘UTR of A. mexicanum embryos.

## Slide 4
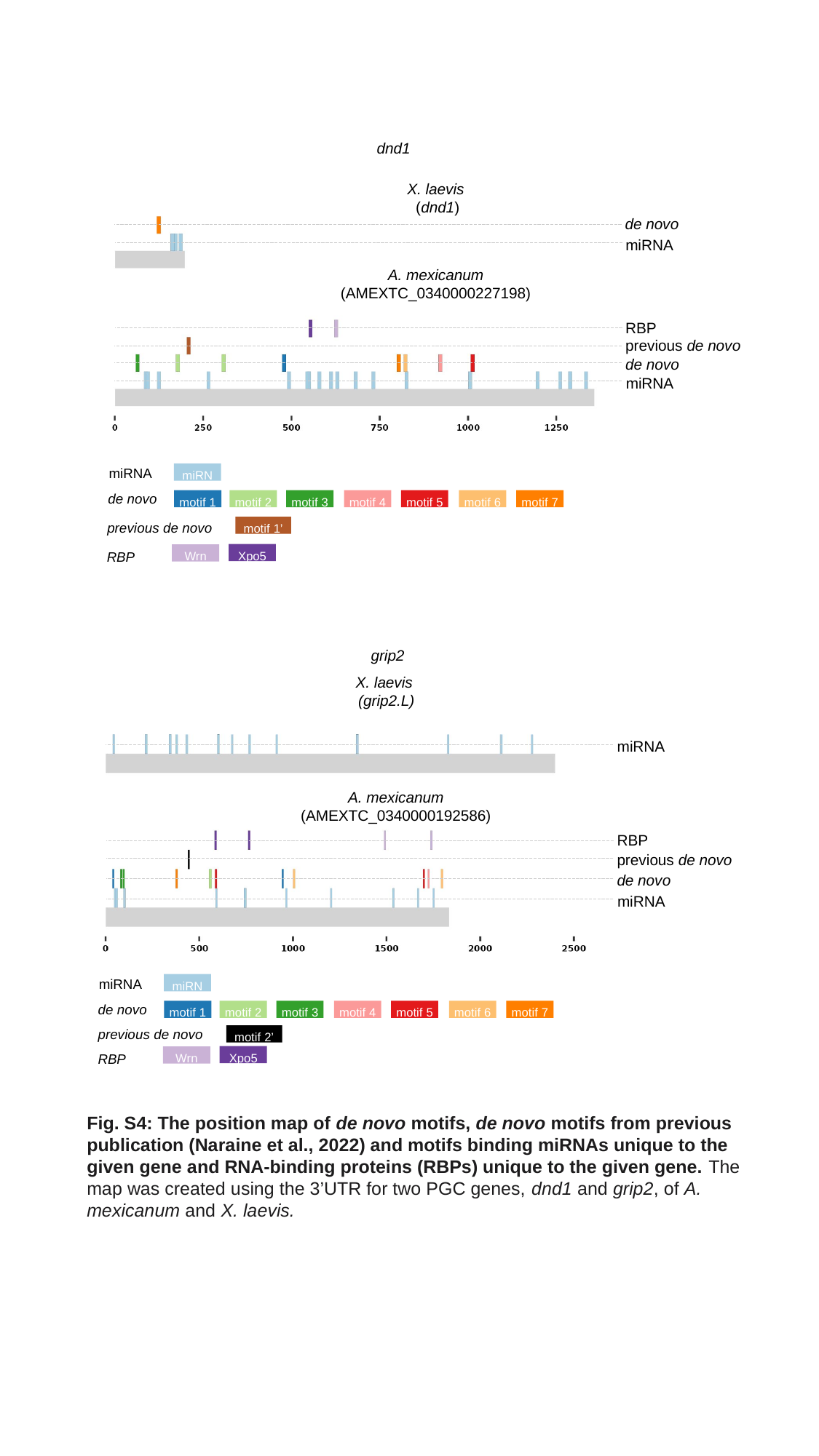

dnd1
X. laevis
(dnd1)
de novo
miRNA
A. mexicanum (AMEXTC_0340000227198)
RBP
previous de novo
de novo
miRNA
miRNA
miRNA
de novo
motif 1
motif 2
motif 3
motif 4
motif 5
motif 6
motif 7
previous de novo
motif 1’
RBP
Xpo5
Wrn
grip2
X. laevis
(grip2.L)
miRNA
A. mexicanum (AMEXTC_0340000192586)
RBP
previous de novo
de novo
miRNA
miRNA
miRNA
de novo
motif 1
motif 2
motif 3
motif 4
motif 5
motif 6
motif 7
previous de novo
motif 2’
RBP
Xpo5
Wrn
Fig. S4: The position map of de novo motifs, de novo motifs from previous publication (Naraine et al., 2022) and motifs binding miRNAs unique to the given gene and RNA-binding proteins (RBPs) unique to the given gene. The map was created using the 3’UTR for two PGC genes, dnd1 and grip2, of A. mexicanum and X. laevis.
