# Supplementary material for: RNA localization during early development of the axolotl
Source: Front Cell Dev Biol. 2023 Oct 19;11:1260795. doi: 10.3389/fcell.2023.1260795 (PMC10620976; doi:10.3389/fcell.2023.1260795)
Supplement: Supplementary file 2 [file DataSheet1.pdf]

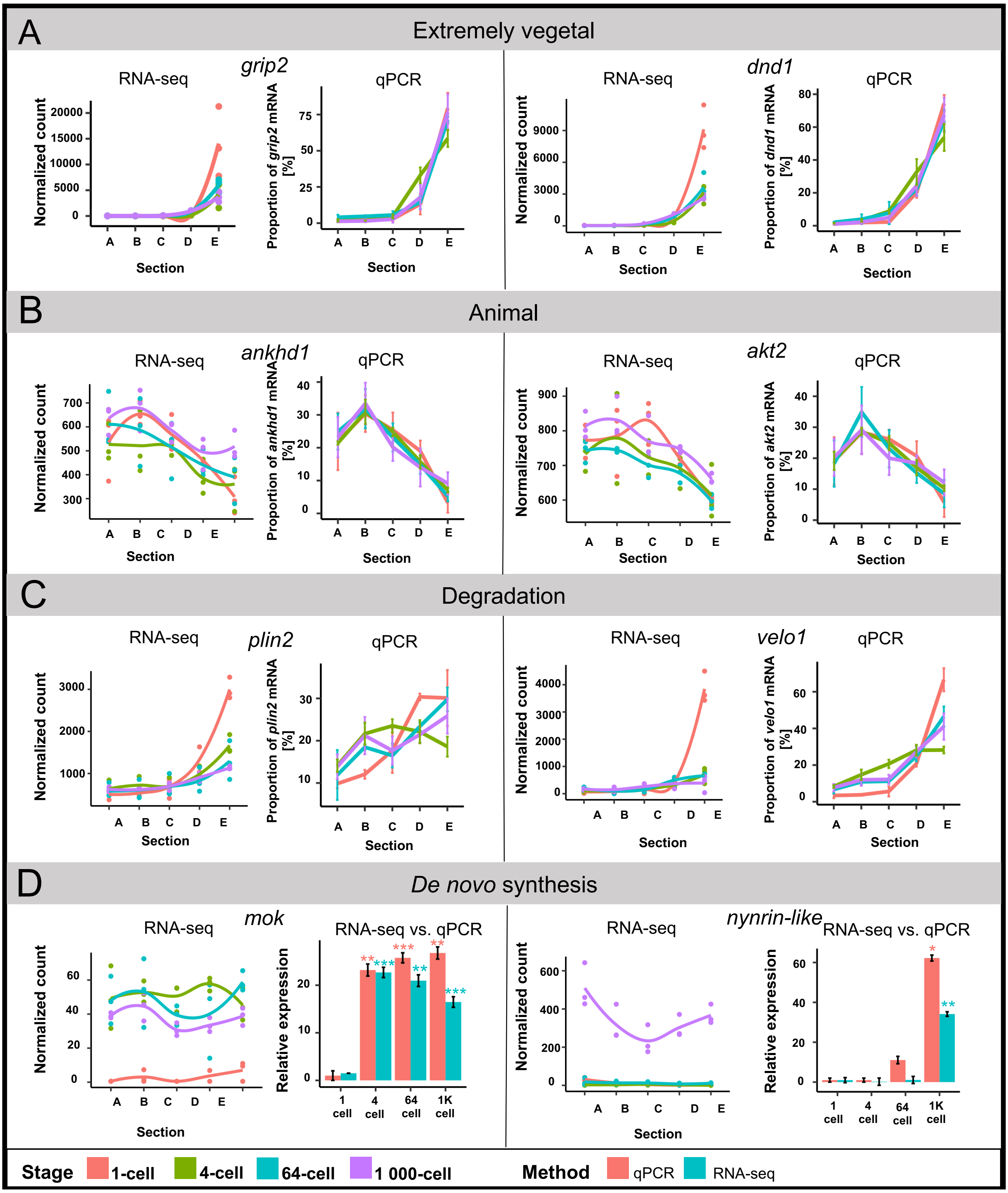

Fig. S1: RNA-seq data validation using RT-qPCR. A – C) RNA-seq represents the averaged z-score expression for the averaged total transcript across the stage replicates. qPCR represents mean proportion of mRNAs for individual genes in particular section. Mean ± .s.d. **D)** RNA-seq - localization profiles generated from RNA-seq data. Line plot represent the averaged z-score expression for the averaged total transcript across the stage replicates. RNA-seq vs. qPCR - the comparison of relative expression using qPCR and RNA-seq. Geometric mean ± g.s.d. The independent samples t-test. N.s . p>0.05, *p<0.05, **p<0.01, ***p<0.001. 3 biological replicates were used.

A

**Profile alteration**	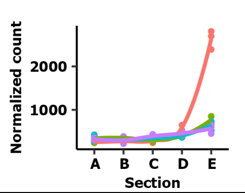	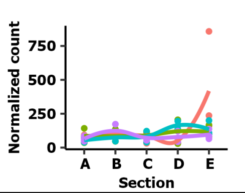	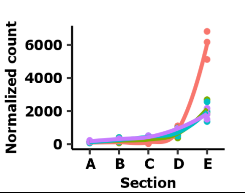	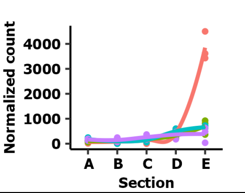	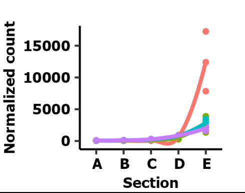
**Gene ID**	AMEXTC_0340000038222	AMEXTC_0340000294788	AMEXTC_0340000183471	AMEXTC_0340000183472	AMEXTC_0340000239076
**Gene symbol**	rbpms2-1	rbpms2-2	velo1-1	velo1-2	nanos1
**Count alteration**	x	x	x	degradation	degradation
**Profile alteration**	x	x	x	x	x
**Experiment**	*in situ;* 3'UTR, 5'UTR motif	x	x	x	3'UTR, 5'UTR motif analysis

**Profile alteration**	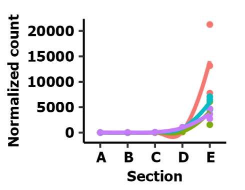	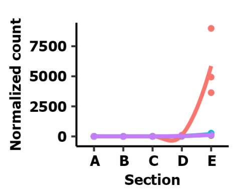	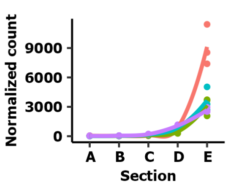	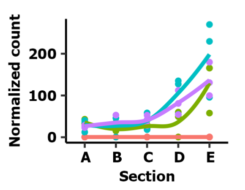
**Gene ID**	AMEXTC_0340000035404	AMEXTC_0340000192586	AMEXTC_0340000227198	AMEXTC_0340000004005
**Gene symbol**	grip2-1	grip2-2	dnd1	x
**Transcript count**	x	degradation	x	*de novo* transcription
**Profile alteration**	Vegetal at the 1-cell stage, gradual decrease in late stages	Vegetal at the 1-cell stage, gradual decrease in late stages	Vegetal at the 1-cell stage, gradual decrease in late stages	Homogeneous/slightly vegetal at the 1-cell stage, vegetal in late stages
**Experiment**	*in situ;* 3'UTR, 5'UTR motif analysis	3'UTR, 5'UTR motif analysis	*in situ;* 3'UTR, 5'UTR motif analysis	*in situ*

B

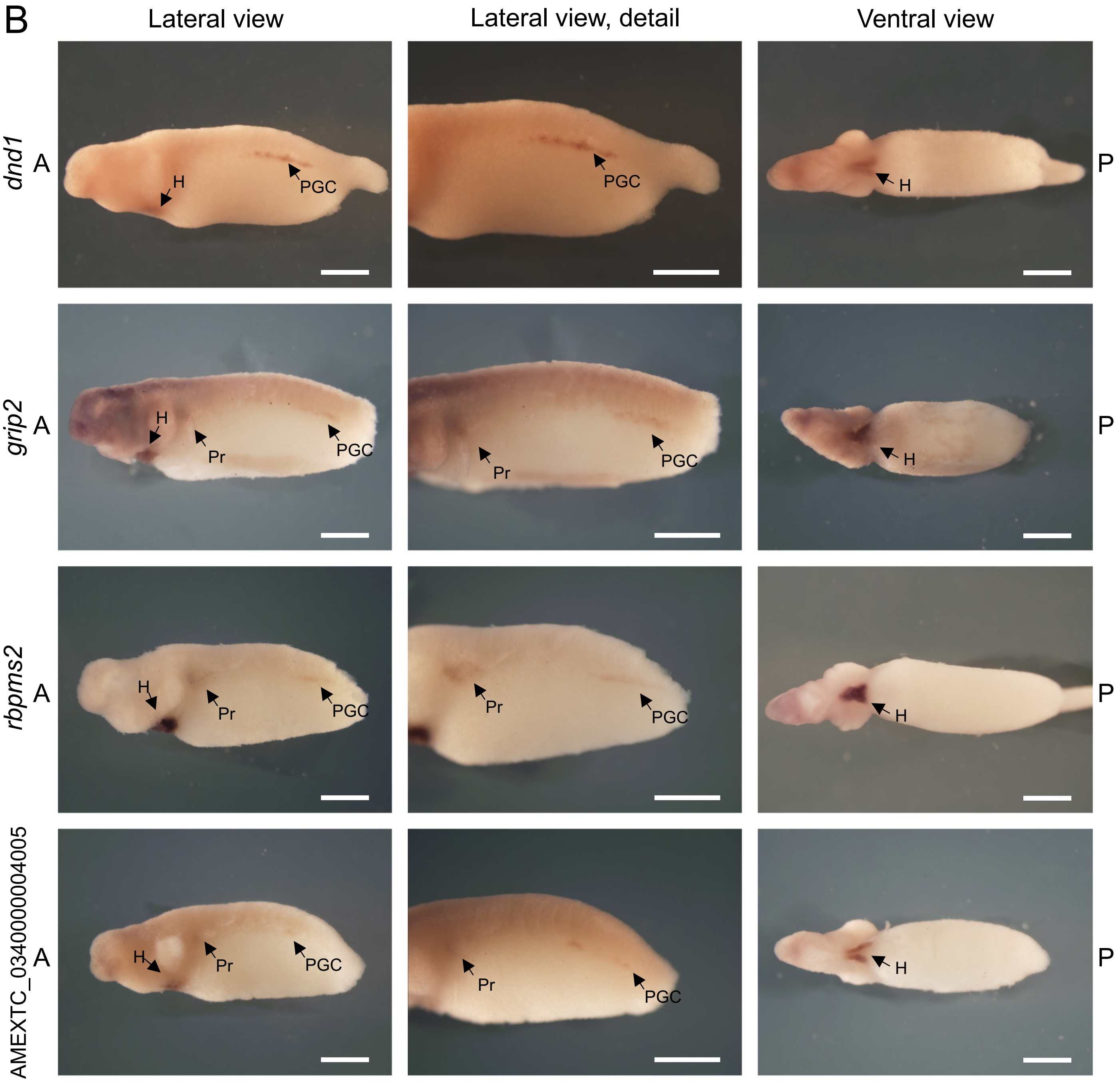

Fig. S2: Primordial germ cell markers in *A. mexicanum*. A) Complete list of primordial germ cell markers. B) Expression patterns of *dnd1, grip2, rbpms2* and AMEXTC_0340000004005 at around embryonic stage 33 (note that the tailbud was lost in some embryos during the in situ hybridization protocol). H – heart; Pr – pronephros; PGC – primordial germ cells. A - anterior, P - posterior. Scale bars = 1 mm.

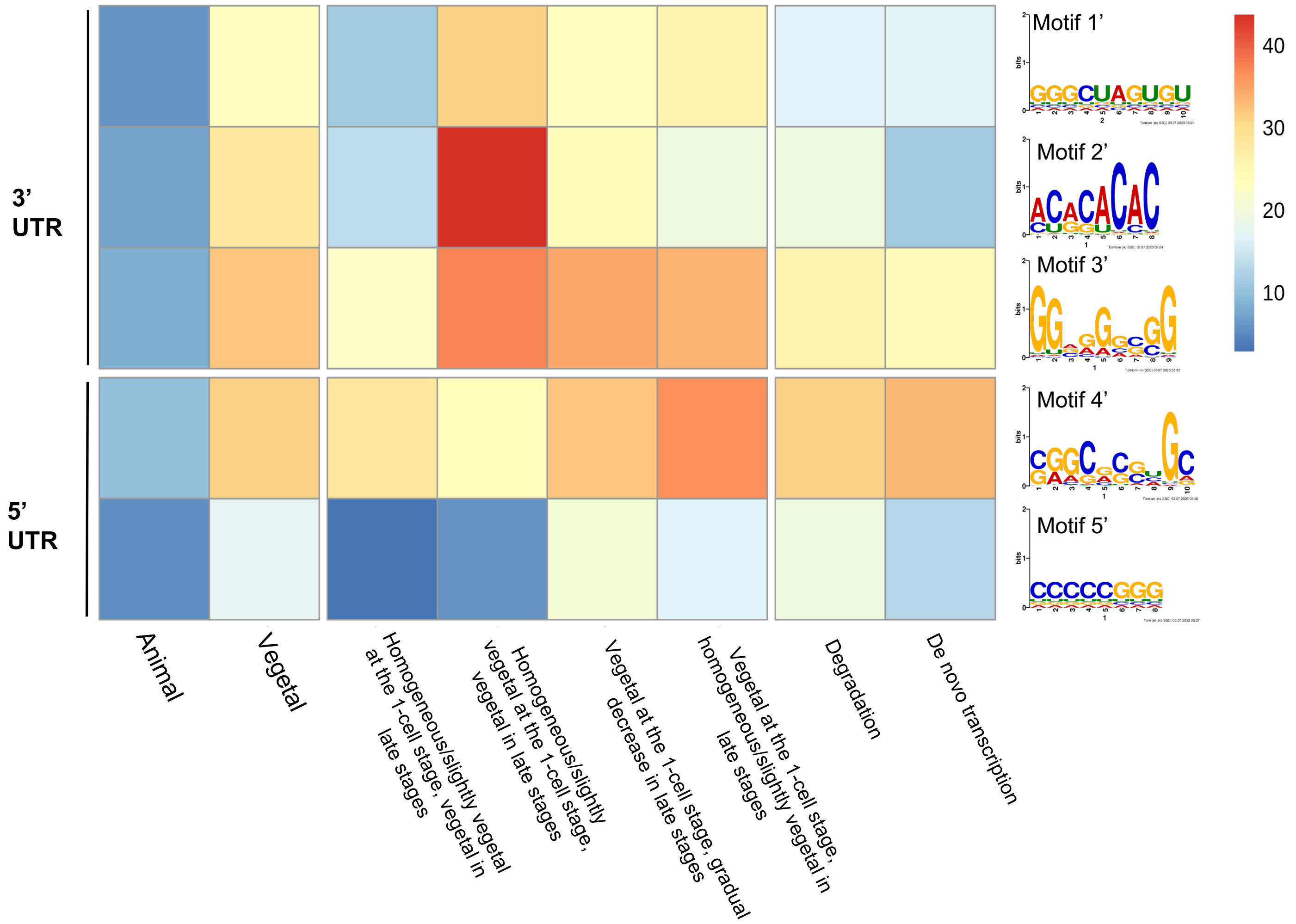

Figure S3: The enrichment of motifs from previous paper (Naraine et al., 2022) within 3' and 5'UTR of *A. mexicanum embryos.*

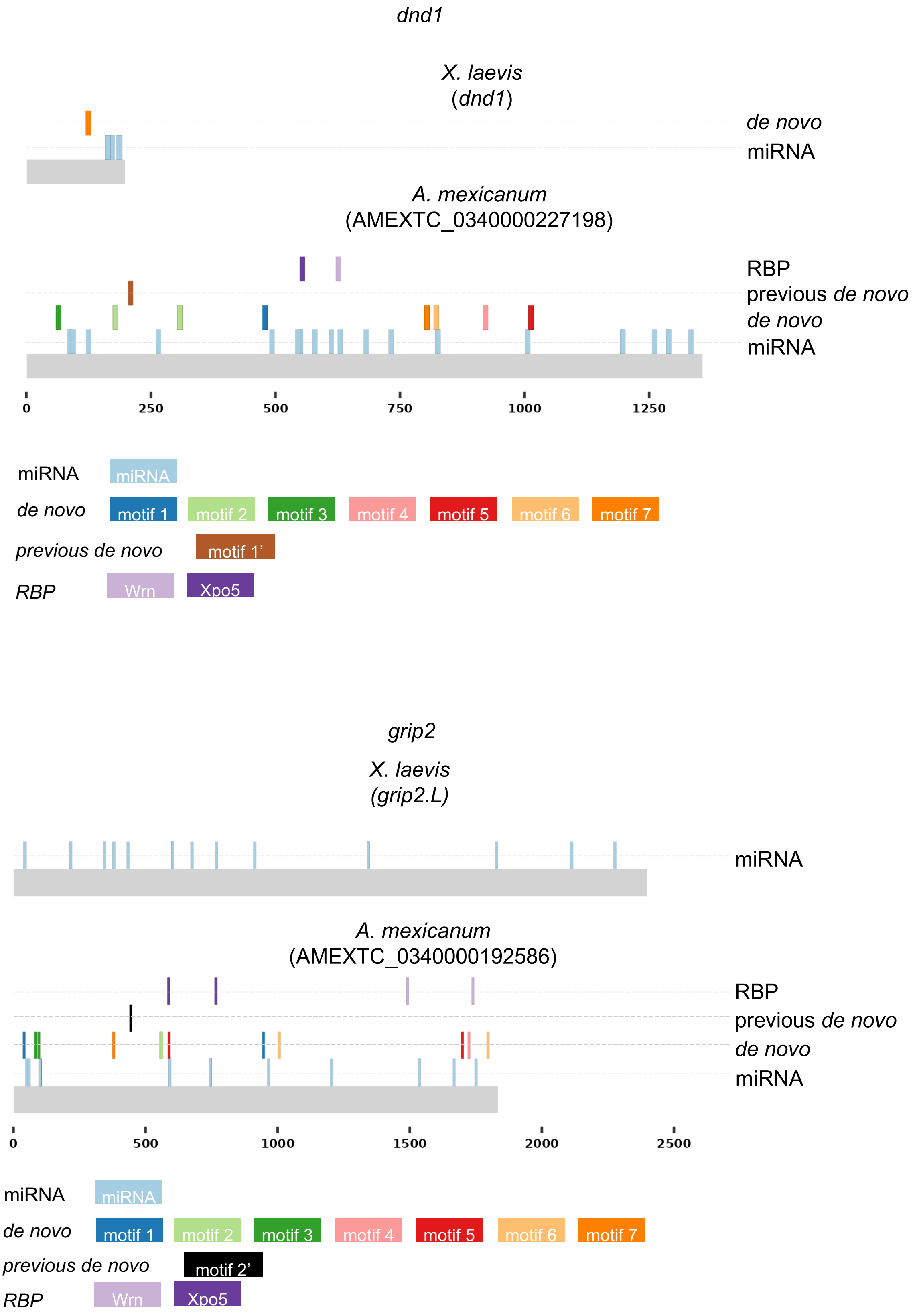

Fig. S4: The position map of *de novo* motifs, *de novo* motifs from previous publication (Naraine et al., 2022) and motifs binding miRNAs unique to the given gene and RNA-binding proteins (RBPs) unique to the given gene. The map was created using the 3’UTR for two PGC genes, *dnd1* and *grip2*, of *A. mexicanum* and *X. laevis.*
